# Discovery of fifteen new geroprotective plant extracts and identification of cellular processes they affect to prolong the chronological lifespan of budding yeast

**DOI:** 10.18632/oncotarget.27615

**Published:** 2020-06-09

**Authors:** Pamela Dakik, Monica Enith Lozano Rodriguez, Jennifer Anne Baratang Junio, Darya Mitrofanova, Younes Medkour, Tala Tafakori, Tarek Taifour, Vicky Lutchman, Eugenie Samson, Anthony Arlia-Ciommo, Belise Rukundo, Éric Simard, Vladimir I. Titorenko

**Affiliations:** ^1^Department of Biology, Concordia University, Montreal, Quebec H4B 1R6, Canada; ^2^Idunn Technologies Inc., Rosemere, Quebec J7A 4A5, Canada

**Keywords:** cellular aging, longevity, gerotargets, geroprotectors, plant extracts

## Abstract

In a quest for previously unknown geroprotective natural chemicals, we used a robust cell viability assay to search for commercially available plant extracts that can substantially prolong the chronological lifespan of budding yeast. Many of these plant extracts have been used in traditional Chinese and other herbal medicines or the Mediterranean and other customary diets. Our search led to a discovery of fifteen plant extracts that significantly extend the longevity of chronologically aging yeast not limited in calorie supply. We show that each of these longevity-extending plant extracts is a geroprotector that decreases the rate of yeast chronological aging and promotes a hormetic stress response. We also show that each of the fifteen geroprotective plant extracts mimics the longevity-extending, stress-protecting, metabolic and physiological effects of a caloric restriction diet but if added to yeast cultured under non-caloric restriction conditions. We provide evidence that the fifteen geroprotective plant extracts exhibit partially overlapping effects on a distinct set of longevity-defining cellular processes. These effects include a rise in coupled mitochondrial respiration, an altered age-related chronology of changes in reactive oxygen species abundance, protection of cellular macromolecules from oxidative damage, and an age-related increase in the resistance to long-term oxidative and thermal stresses.

## INTRODUCTION

The budding yeast *Saccharomyces cerevisiae* is a widely used model organism in aging research because it offers three significant advantages in studying mechanisms of aging and longevity [1–5]. First, *S. cerevisiae* has relatively short and easily measurable replicative and chronological lifespans. Second, the *S. cerevisiae* genome has been completely sequenced and many strain collections for yeast genome interrogation are commercially available. Third, *S. cerevisiae* is amenable to comprehensive molecular analyses that have been used to uncover mechanisms of various cell biological processes [4, 6–10]. Because of these advantages, studies in *S. cerevisiae* discovered many genes, signaling pathways and chemical compounds that, following their discovery in budding yeast, were implicated in aging and longevity in organisms across an evolutionary tree [4, 5, 8, 11–15]. It is therefore commonly believed that the major aspects and underlying mechanisms of aging and aging-associated pathology have been conserved throughout evolution [8, 14–32].

Our research aims to understand mechanisms through which certain chemical compounds of plant origin act as geroprotectors capable of delaying chronological aging and postponing aging-associated pathology in budding yeast. Our recent screen of a collection of thirty-five plant extracts (PEs) had identified six PEs that can prolong chronological lifespan (CLS) and delay chronological aging in *S. cerevisiae* [33]. We found that in *S. cerevisiae,* the six PEs exhibit different effects on cellular processes known to define longevity in eukaryotes across species [33]. These effects of the six aging-delaying PEs included an increase in mitochondrial respiration and membrane potential, a moderate but significant rise or decline in cellular reactive oxygen species (ROS), a weakening of oxidative damage to cellular proteins, lipids and DNA, an augmentation of cell resistance to long-term oxidative and thermal stresses, and an enhancement of neutral lipid lipolysis in lipid droplets [33]. We also revealed that the six PEs extend yeast CLS through different signaling pathways and protein kinases converged into a network; this network is known to define the rate of chronological aging in *S. cerevisiae* and to regulate longevity in other eukaryotic organisms [34]. The network integrates the pro-aging TORC1 (target of rapamycin complex 1) pathway, the pro-aging PKA (protein kinase A) pathway, the pro-aging PKH1/2 (Pkb-activating kinase homolog) pathway, the anti-aging SNF1 (sucrose non-fermenting) pathway, the anti-aging ATG (autophagy) pathway, the pro-aging serine/threonine-protein kinase Sch9 and the anti-aging serine/threonine-protein kinase Rim15 (regulator of *IME2*) [34]. We provided evidence that pairwise combinations of the six PEs display synergistic effects on the delay of yeast chronological aging only if each of the PEs comprising a combination targets a different signaling pathway or protein kinase of longevity regulation [35]. Moreover, we described three different mechanisms through which one of the six PEs, which is called PE21, can delay yeast chronological aging and extend yeast longevity [36].

The objective of the present study was to search for previously unknown aging-delaying (geroprotective) PEs. To attain this objective, we conducted a new screen of many extracts from plants used in traditional Chinese and other herbal medicines or the Mediterranean and other diets. Our screen discovered fifteen new geroprotective PEs that extend yeast CLS. We show that each of these new aging-delaying PEs decreases the rate of yeast chronological aging, stimulates a hormetic stress response and regulates a distinct set of longevity-defining cellular processes.

## RESULTS

### Identification of new PEs that prolong the longevity of chronologically aging budding yeast

In search of new geroprotective PEs, we performed a screen of fifty-three commercially available PEs. The origin and properties of these PEs are shown in Supplementary Table 1. These PEs are believed to have positive effects on human health, and many of them have been used in traditional Chinese and other herbal medicines or the Mediterranean and other long-established diets.

To conduct the screen, we exploited a robust clonogenic cell viability assay for measuring yeast CLS [33]. In this assay, the wild-type (WT) strain BY4742 was cultured in the synthetic minimal YNB medium initially containing 2% (w/v) glucose, as described in Materials and Methods. Cells of budding yeast cultured under such non-caloric restriction (non-CR) conditions are known to age chronologically faster than the ones cultured under CR conditions on 0.2% (w/v) or 0.5% (w/v) glucose [1, 3, 4, 33].

At the time of cell inoculation into the culturing medium, we added each of the assessed PEs at a final concentration ranging from 0.02% (w/v) to 1.0% (w/v). We found that PE40, PE41, PE44, PE50, PE53, PE66, PE73, PE84, PE86 and PE87 do not affect the mean and maximum CLS of WT yeast if exogenously supplemented within this wide range of initial concentrations (Supplementary Figures 1–7). In contrast, PE38, PE43, PE45, PE46, PE48, PE49, PE51, PE52, PE54-PE58, PE60-PE63, PE65, PE67, PE70, PE71, PE74, PE76, PE80, PE82, PE85, PE88 and PE89 were cytotoxic at certain concentrations; they decreased the mean and/or maximum CLS of WT yeast if used at the final concentrations in the 0.1 (w/v) to 1.0% (w/v) range (Supplementary Figures 1–7).

**Figure 1:**
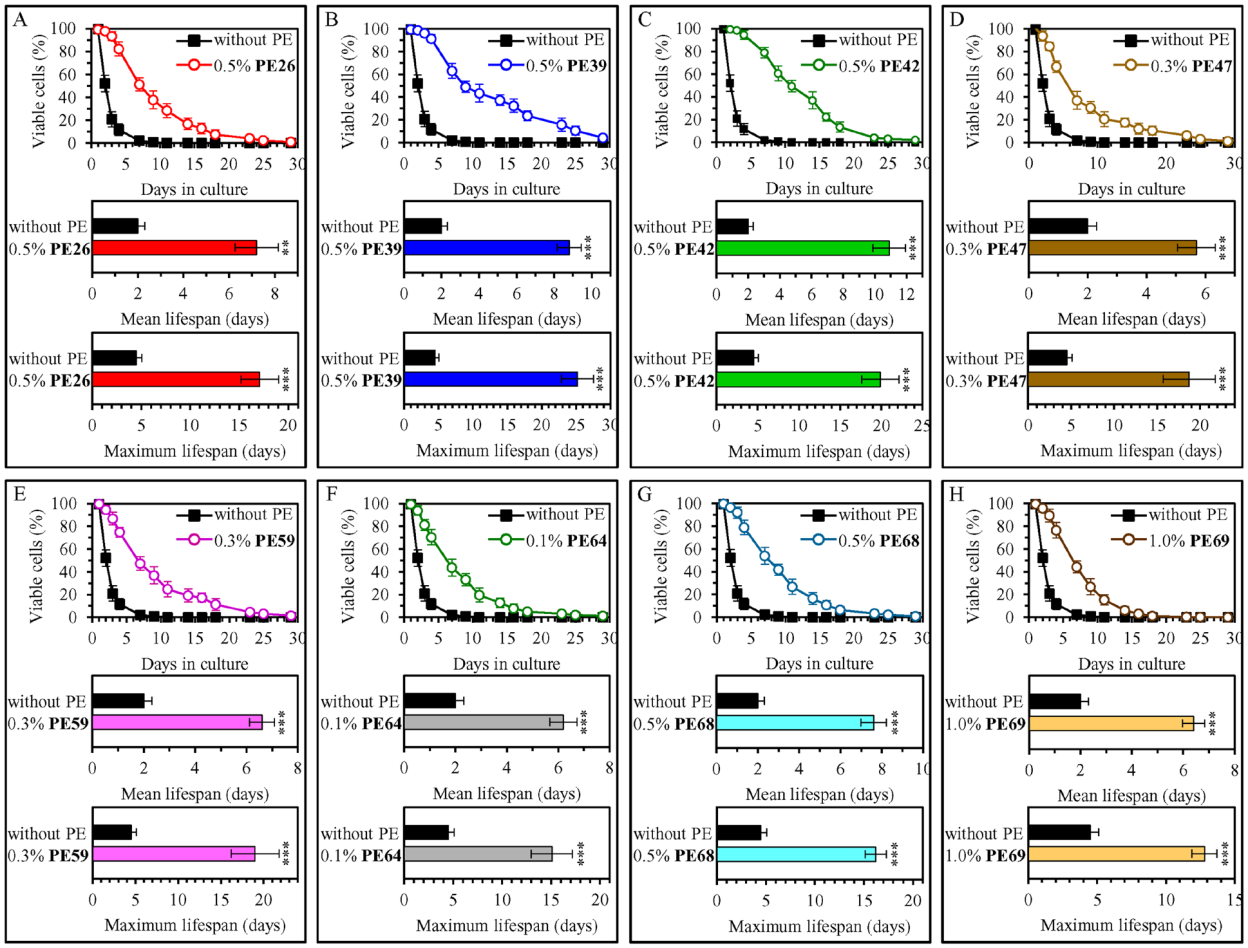
0.5% (w/v) PE26, 0.5% (w/v) PE39, 0.5% (w/v) PE42, 0.3% (w/v) PE47, 0.3% (w/v) PE59, 0.1% (w/v) PE64, 0.5% (w/v) PE68 and 1.0% (w/v) PE69 exhibit the highest extending effects on the chronological lifespan (CLS) of wild-type (WT) yeast cultured under non-CR conditions on 2% (w/v) glucose. WT cells were cultured in the synthetic minimal YNB medium initially containing 2% (w/v) glucose, in the presence of a PE or its absence. In the cultures supplemented with a PE, ethanol was used as a vehicle at a final concentration of 2.5% (v/v). In the same experiment, WT cells were also subjected to ethanol-mock treatment by being cultured in the synthetic minimal YNB medium initially containing 2% (w/v) glucose and 2.5% (v/v) ethanol. Survival curves (the upper panels in **A**–**H**) and the mean and maximum lifespans (the lower two panels in A–H) of chronologically aging WT cells cultured without a PE (cells were subjected to ethanol-mock treatment) or with a PE (which was added at the concentration optimal for CLS extension) are shown. Data are presented as means ± SEM (*n* = 6). In the upper panels in A–H, CLS extension was significant for each of the PEs tested (*p* < 0.05; the *p* values for comparing each pair of survival curves were calculated using the logrank test as described in Materials and Methods). In the lower two panels in A–H, ^*^ *p* < 0.05, ^**^ *p* < 0.01, ^***^ *p* < 0.001; the *p* values for comparing the means of two in groups were calculated using an unpaired two-tailed *t* test as described in Materials and Methods). Data for mock-treated WT cells are replicated in graphs A–H of this Figure, graphs A–G of Figure 2 and Supplementary Figures 1–7. Data for WT cells cultured with a PE added at the concentration optimal for CLS extension are replicated in Supplementary Figure 1 (for 0.5% (w/v) PE26, 0.5% (w/v) PE39 and 0.5% (w/v) PE42), Supplementary Figure 2 (for 0.3% (w/v) PE47), Supplementary Figure 3 (for 0.3% (w/v) PE59), Supplementary Figure 4 (for 0.1% (w/v) PE64 and 0.5% (w/v) PE68) and Supplementary Figure 5 (for 1.0% (w/v) PE69).

**Figure 2:**
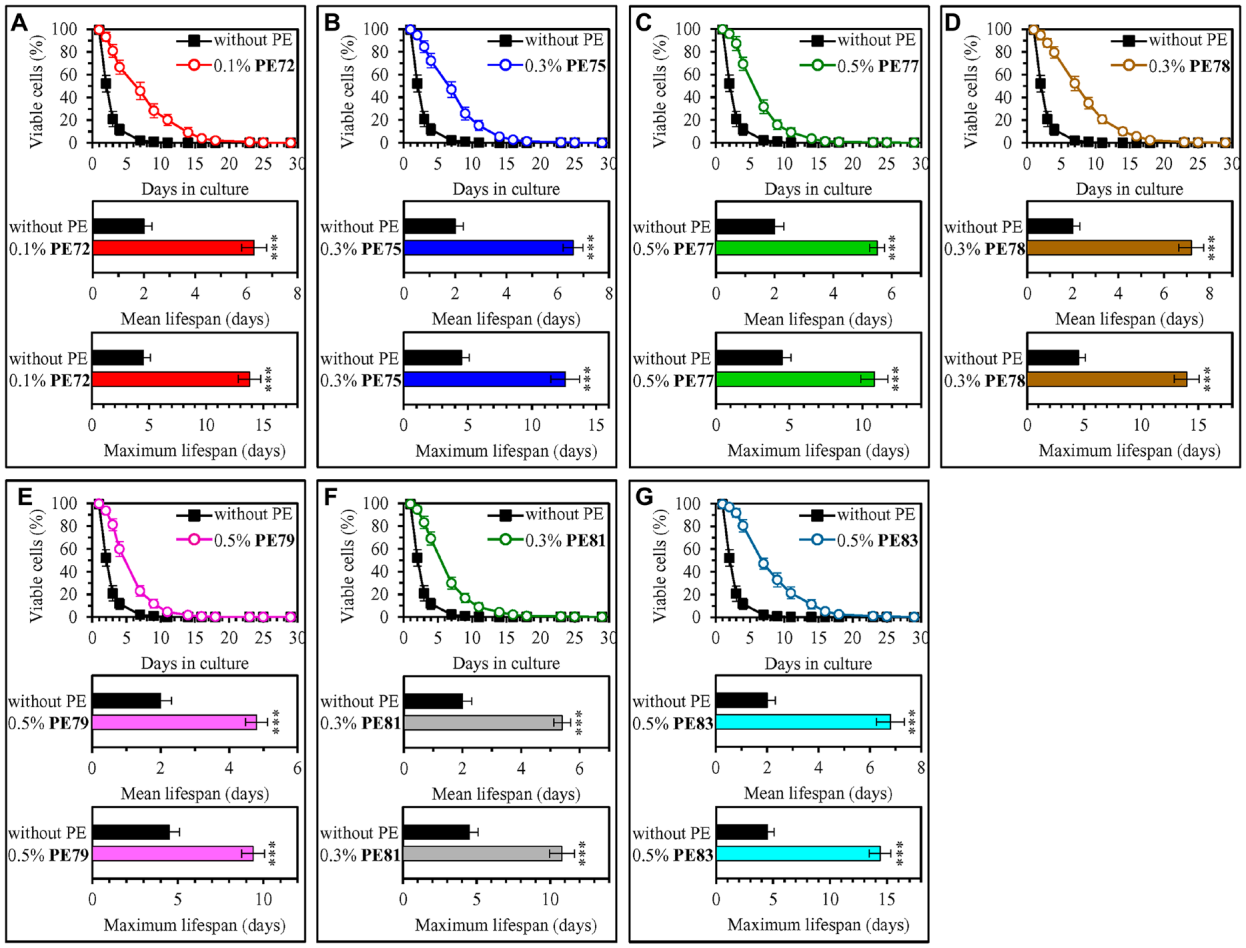
0.1% (w/v) PE72, 0.3% (w/v) PE75, 0.5% (w/v) PE77, 0.3% (w/v) PE78, 0.5% (w/v) PE79, 0.3% (w/v) PE81 and 0.5% (w/v) PE83 exhibit the highest extending effects on the CLS of WT yeast cultured under non-CR conditions on 2% (w/v) glucose. WT cells were cultured in the synthetic minimal YNB medium initially containing 2% (w/v) glucose, in the presence of a PE or its absence. In the cultures supplemented with a PE, ethanol was used as a vehicle at a final concentration of 2.5% (v/v). In the same experiment, WT cells were also subjected to ethanol-mock treatment by being cultured in the synthetic minimal YNB medium initially containing 2% (w/v) glucose and 2.5% (v/v) ethanol. Survival curves (the upper panels in **A**–**G**) and the mean and maximum lifespans (the lower two panels in A–G) of chronologically aging WT cells cultured without a PE (cells were subjected to ethanol-mock treatment) or with a PE (which was added at the concentration optimal for CLS extension) are shown. Data are presented as means ± SEM (*n* = 6). In the upper panels in A–G, CLS extension was significant for each of the PEs tested (*p* < 0.05; the *p* values for comparing each pair of survival curves were calculated using the logrank test as described in Materials and Methods). In the lower two panels in A–G, ^*^ *p* < 0.05, ^**^ *p* < 0.01, ^***^ *p* < 0.001; the *p* values for comparing the means of two in groups were calculated using an unpaired two-tailed *t* test as described in Materials and Methods). Data for mock-treated WT cells are replicated in graphs A–G of this Figure, graphs A–H of Figure 1 and Supplementary Figures 1–7. Data for WT cells cultured with a PE added at the concentration optimal for CLS extension are replicated in Supplementary Figure 4 (for 0.1% (w/v) PE72 and 0.3% (w/v) PE75) and Supplementary Figure 6 (for 0.5% (w/v) PE77, 0.3% (w/v) PE78, 0.5% (w/v) PE79, 0.3% (w/v) PE81 and 0.5% (w/v) PE83).

Our screen revealed that fifteen of fifty-three tested PEs statistically significantly increase the mean and maximum CLS of WT yeast cultured under non-CR conditions on 2% (w/v) glucose (Figures 1 and 2; Supplementary Figures 1–6). Each of these fifteen PEs extended the longevity of chronologically aging WT yeast if used within a specific concentration range and exhibited the highest longevity-extending effect at a certain concentration within this range (Supplementary Figures 1–6). The following PEs exhibited the highest longevity-extending effect under non-CR conditions of cell culturing: 0.5% (w/v) PE26 from berries of *Serenoa repens* (Figure 1A, Supplementary Figure 1), 0.5% (w/v) PE39 from aerial parts of *Hypericum perforatum* (Figure 1B, Supplementary Figure 1), 0.5% (w/v) PE42 from leaves of *Ilex paraguariensis* (Figure 1C, Supplementary Figure 1), 0.3% (w/v) PE47 from leaves of *Ocimum tenuiflorum* (Figure 1D, Supplementary Figure 2), 0.3% (w/v) PE59 from the whole plant of *Solidago virgaurea* (Figure 1E, Supplementary Figure 3), 0.1% (w/v) PE64 from fruits of *Citrus sinensis* (Figure 1F, Supplementary Figure 4), 0.5% (w/v) PE68 from the whole plant of *Humulus lupulus* (Figure 1G, Supplementary Figure 4), 1.0% (w/v) PE69 from grape skins of *Vitis vinifera* (Figure 1H, Supplementary Figure 5), 0.1% (w/v) PE72 from the whole plant of *Andrographis paniculata* (Figure 2A, Supplementary Figure 5), 0.3% (w/v) PE75 from roots of *Hydrastis canadensis* (Figure 2B, Supplementary Figure 5), 0.5% (w/v) PE77 from seeds of *Trigonella foenum-graecum* (Figure 2C, Supplementary Figure 6), 0.3% (w/v) PE78 from root barks of *Berberis vulgaris* (Figure 2D, Supplementary Figure 6), 0.5% (w/v) PE79 from leaves, flowers and stems of *Crataegus monogyna* (Figure 2E, Supplementary Figure 6), 0.3% (w/v) PE81 from leaves of *Taraxacum erythrospermum* (Figure 2F, Supplementary Figure 6), and 0.5% (w/v) PE83 from the whole plant of of *Ilex paraguariensis* (Figure 2G, Supplementary Figure 6).

We found that none of the fifteen longevity-extending PEs displays a statistically significant effect on glucose consumption during culturing of WT cells under non-CR conditions on 2% (w/v) glucose (Supplementary Figure 8). This finding shows that each of these PEs prolongs the longevity of chronologically aging yeast not because it alters the concentration of exogenous glucose and, thus, not because it affects the metabolic rate of this major source of carbon and energy. We also found that none of the fifteen longevity-extending PEs exhibits a statistically significant effect on the growth rate and maximum cell yield of WT yeast cultures under non-CR conditions (Supplementary Figure 9). Based on this observation, we concluded that each of them extends the longevity of chronologically aging yeast not because it slows cell proliferation and, thus, not because it desensitizes yeast to harmful chemical compounds produced when cells proliferate.

### Each of the fifteen longevity-prolonging PEs mimics longevity extension by CR

CR without malnutrition is a low-calorie dietary regimen that extends lifespan in many evolutionarily distant organisms and improves healthspan in laboratory rodents and rhesus monkeys [4, 8, 37–39]. Certain natural chemicals and synthetic drugs have been shown to elicit the CR-like lifespan-extending and healthspan-improving effects even under non-CR conditions (i. e., when calorie supply is not limited) [40–45]. These natural and synthetic chemical compounds are called CR mimetics (CRMs) if they not only extend longevity under non-CR conditions but also if they exhibit three other effects. First, CRMs do not impair food intake. Second, CRMs have CR-like effects on metabolism and physiology. Third, akin to CR, CRMs decrease the susceptibility to diverse stresses [41, 44]. In the present study we found that each of the fifteen longevity-extending PEs increases yeast CLS under non-CR conditions on 2% (w/v) glucose (Figures 1 and 2; Supplementary Figures 1–6) and none of them compromises glucose intake during culturing under these conditions (Supplementary Figure 8). Thus, it seems that all these PEs are CRMs. This conclusion is further supported by our observations that each of the fifteen longevity-extending PEs exhibits CR-like effects on several aspects of cell metabolism and stress resistance (see below).

Of note, we previously reported that if the CR diet is administered by culturing yeast in the YNB medium initially containing 0.5% (w/v) glucose, it significantly increases both the mean and maximum CLS of *S. cerevisiae* [33]. In the present study, we investigated how each of the fifteen PEs that extends longevity under non-CR conditions influences the longevity of yeast cultured under CR conditions on 0.5% (w/v) glucose. We found that eight of the fifteen PEs that prolong the longevity of chronologically aging yeast under non-CR conditions do not increase either the mean or the maximum CLS of *S. cerevisiae* under CR conditions (Supplementary Figures 10 and 11). These PEs included 0.3% (w/v) PE47 (Supplementary Figure 10D), 0.1% (w/v) PE64 (Supplementary Figure 10F), 1.0% (w/v) PE69 (Supplementary Figure 10H), 0.1% (w/v) PE72 (Supplementary Figure 11A), 0.3% (w/v) PE75 (Supplementary Figure 11B), 0.5% (w/v) PE77 (Supplementary Figure 11C), 0.5% (w/v) PE79 (Supplementary Figure 11E) and 0.3% (w/v) PE81 (Supplementary Figure 11F). It seems conceivable, therefore, that each of these eight PEs increases yeast CLS because it modulates the same or highly overlapping sets of longevity-defining cellular processes under both CR and non-CR conditions. Some of these processes can be suppressed under non-CR conditions, whereas others can operate as cellular housekeeping processes that are not regulated in a CR-dependent manner. The relative contributions of the CR-regulated and housekeeping (i. e., not regulated by CR) cellular processes to the process of yeast chronological aging need to be addressed in the future.

We also revealed that seven of the fifteen PEs that extend yeast longevity under non-CR conditions also increase both the mean and maximum CLS of *S. cerevisiae* under CR conditions (Supplementary Figures 10 and 11). 0.5% (w/v) PE26 (Supplementary Figure 10A), 0.5% (w/v) PE39 (Supplementary Figure 10B), 0.5% (w/v) PE42 (Supplementary Figure 10C), 0.3% (w/v) PE59 (Supplementary Figure 10E), 0.5% (w/v) PE68 (Supplementary Figure 10G), 0.3% (w/v) PE78 (Supplementary Figure 11D) and 0.5% (w/v) PE83 (Supplementary Figure 11G) were among these PEs. Therefore, we hypothesize that each of these seven PEs increases yeast CLS under both CR and non-CR conditions because it targets both CR-regulated and housekeeping (i.e., not regulated by CR) cellular processes.

**Figure 3:**
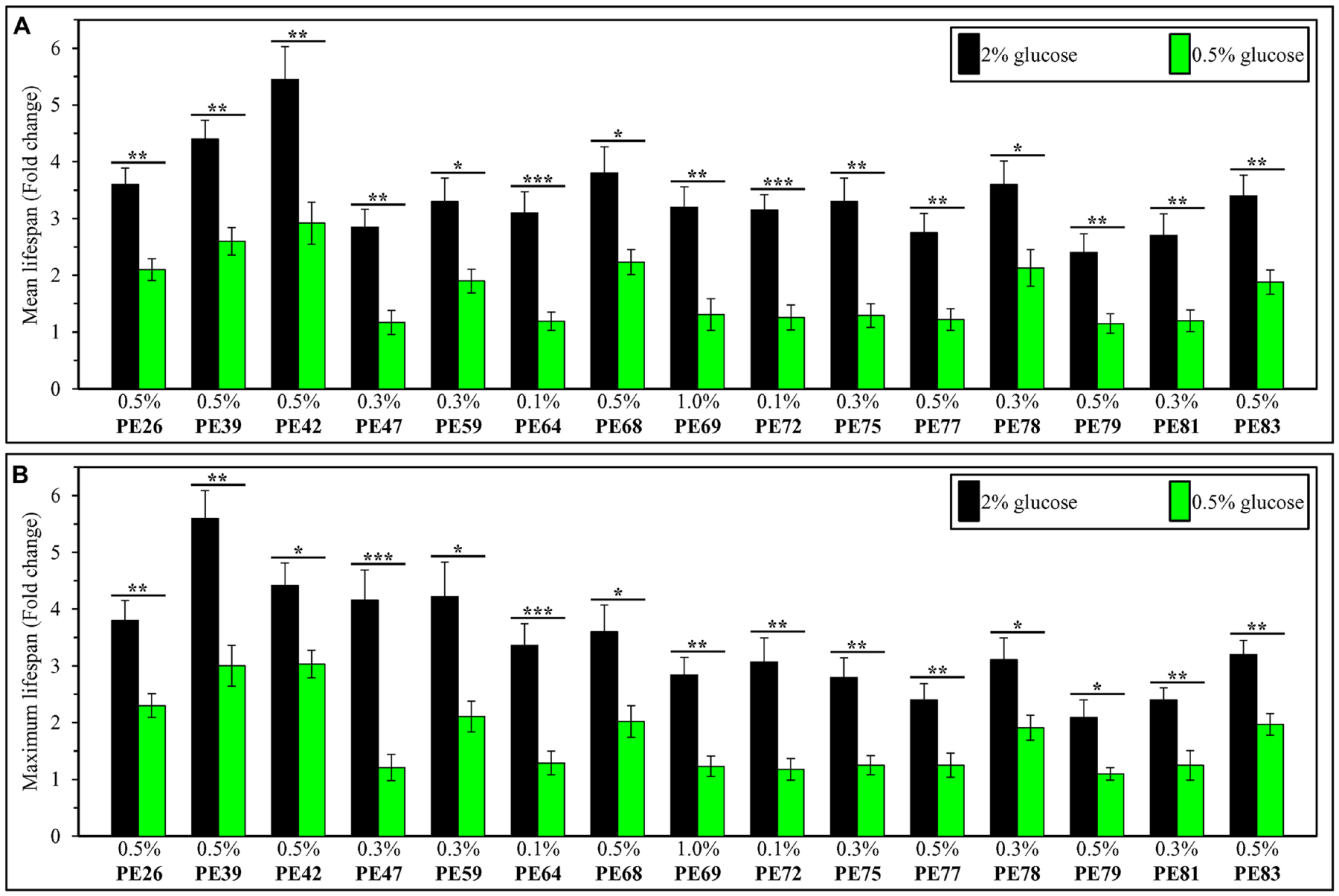
Each of the fifteen PEs extends the longevity of chronologically aging yeast under non-CR conditions on 2% (w/v) glucose significantly more efficiently than it does under CR conditions on 0.5% (w/v) glucose. WT cells were cultured in the synthetic minimal YNB medium initially containing 2% (w/v) or 0.5% (w/v) glucose, in the presence of a PE or its absence. In the cultures supplemented with a PE, ethanol was used as a vehicle at a final concentration of 2.5% (v/v). In the same experiment, WT cells were also subjected to ethanol-mock treatment by being cultured in the synthetic minimal YNB medium initially containing 0.5% (w/v) or 2% (w/v) glucose and 2.5% (v/v) ethanol. The extent to which each of the PE tested increases the mean (**A**) and maximum (**B**) CLS under non-CR and CR conditions was calculated based on the data presented in Figures 1 and 2, and Supplementary Figures 10 and 11. ^*^ *p* < 0.05, ^**^ *p* < 0.01, ^***^ *p* < 0.001; the *p* values for comparing the means of two in groups were calculated using an unpaired two-tailed *t* test as described in Materials and Methods.

We then compared the efficiency with which each of the fifteen PEs increases yeast CLS under non-CR conditions to that under CR conditions. Our comparison revealed that each of these PEs extends the longevity of chronologically aging yeast under non-CR conditions significantly more efficiently than it does under CR conditions (Figure 3). This finding shows that each of the fifteen PEs is a more effective longevity-prolonging intervention in chronologically aging yeast not-limited in calorie supply than it is in yeast placed on a CR diet.

### Each of the fifteen longevity-prolonging PEs is a geroprotector that extends the longevity of chronologically aging yeast because it decreases the rate of aging and stimulates a hormetic stress response

The rate of biological aging at the demographic level depends on the health of a population and can be determined by measuring an age-specific mortality rate of this population [46–50]. The mortality rates of evolutionarily distant organisms rise with age [46, 48–51]. The Gompertz mortality function equation can describe this age-related rise in the mortality rate; this equation can be graphically presented as mortality rate data plotted on a semi-log scale against biological age [46, 48, 50–52]. Geroprotective interventions (also known as geroprotectors) can extend the longevity of organisms across phyla by causing three different effects on the Gompertz mortality function. Some geroprotectors can lower a so-called “baseline” mortality rate by eliciting an equal decline in the mortality rate at any biological age, without affecting a slope of the Gompertz mortality rate [47, 48, 50, 53, 54]. This slope is known as the coefficient *G* of the age-specific mortality rate; it is inversely proportional to the rate of biological aging [47, 48, 50, 53, 54]. Other geroprotectors can decrease the rate of biological aging because they lower the value of *G*, thus raising the value of the mortality rate doubling time (MRDT; MRDT = 0.693/*G*) [47, 48, 50, 55, 56]. The longevity-extending effects of some other geroprotective interventions can represent a combination of both the drop in the baseline mortality rate and the decline in the value of *G* (which raises the value of MRDT) [46–48, 50, 54].

**Figure 4:**
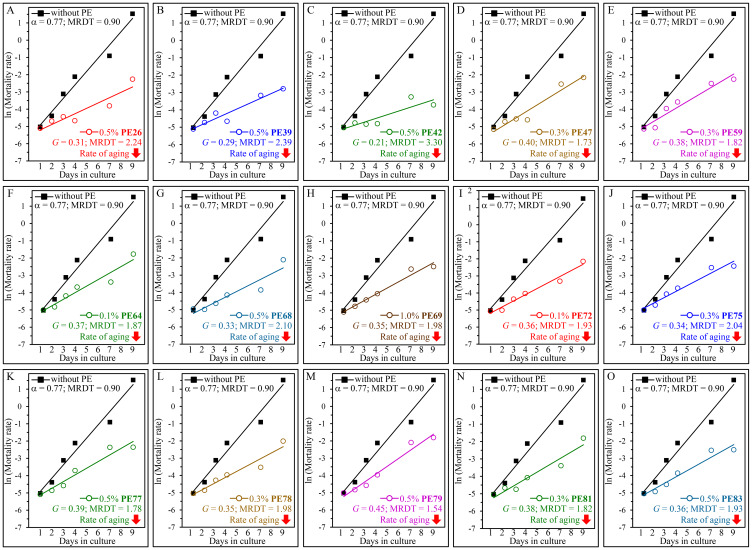
Each of the fifteen PEs extends the longevity of chronologically aging yeast because it decreases the rate of aging but not because it lowers the baseline mortality rate. WT cells were cultured in the synthetic minimal YNB medium initially containing 2% (w/v) glucose, in the presence of a PE or its absence. The following PEs were added to the culture: 0.5% (w/v) PE26 (**A**), 0.5% (w/v) PE39 (**B**), 0.5% (w/v) PE42 (**C**), 0.3% (w/v) PE47 (**D**), 0.3% (w/v) PE59 (**E**), 0.1% (w/v) PE64 (**F**), 0.5% (w/v) PE68 (**G**), 1.0% (w/v) PE69 (**H**), 0.1% (w/v) PE72 (**I**), 0.3% (w/v) PE75 (**J**), 0.5% (w/v) PE77 (**K**), 0.3% (w/v) PE78 (**L**), 0.5% (w/v) PE79 (**M**), 0.3% (w/v) PE81 (**N**) or 0.5% (w/v) PE83 (**O**). In the cultures supplemented with a PE, ethanol was used as a vehicle at a final concentration of 2.5% (v/v). In the same experiment, WT cells were also subjected to ethanol-mock treatment by being cultured in the synthetic minimal YNB medium initially containing 2% (w/v) glucose and 2.5% (v/v) ethanol. Survival curves shown in Figures 1 and 2 were used to calculate the age-specific mortality rates of chronologically aging WT yeast populations cultured without a PE (cells were subjected to ethanol-mock treatment) or with a PE (which was added at the concentration optimal for CLS extension). The natural logarithms of the mortality rate values for each time point were plotted against days of cell culturing. The values of the age-specific mortality rates, Gompertz slope (also known as the mortality rate coefficient *G)* and mortality rate doubling time (MRDT) were calculated as described in Materials and Methods. Each of the fifteen longevity-extending PEs caused a substantial decline in the value of G and a considerable rise in the value of MRDT.

We sought to investigate whether each of the fifteen PEs extends yeast longevity by lowering the baseline mortality rate, decreasing the rate of biological aging or by altering both these rates. Therefore, we conducted the Gompertz mortality rate analysis of WT cells under non-CR conditions that were either treated with one of these PEs or subjected to mock treatment. We found the following: 1) none of the fifteen longevity-prolonging PEs affects the baseline mortality rate, and 2) each of them elicits a decline in the coefficient *G* of the age-specific mortality rate and causes a rise in the value of MRDT (Figure 4). Based on these observations, we concluded that each of these PEs is a geroprotector that lengthens the longevity of chronologically aging yeast because it lowers the rate of aging but not because it decreases the baseline mortality rate.

Our data allow us to conclude that each of the fifteen longevity-prolonging PEs slows yeast chronological aging because it decreases both the extrinsic and the intrinsic rates of aging. This conclusion is based on our findings that each of these PEs extends both the mean and maximum CLS of yeast (Figures 1 and 2). The mean lifespans of evolutionarily distant organisms are thought to depend on certain environmental (extrinsic) factors to which cells are exposed before they enter the quiescent or senescent state [24, 48, 57, 58]. In contrast, the maximum lifespans of organisms across species are considered to rely on certain cellular and organismal longevity modifiers that operate after cells enter the quiescent or senescent state [48, 52, 57, 58, 59].

Our data also show that the ability of each of the fifteen longevity-prolonging PEs to decelerate yeast chronological aging correlates with (and is possibly caused by) its ability to elicit a “hormetic” stress response. A characteristic feature of such a response is a nonlinear and biphasic (i. e., inverted U-shaped or J-shaped) dose-response curve [21, 60–63]. As we found, the curves that reflect relationships between PE concentrations and mean or maximum yeast CLS are inverted U-shaped or J-shaped for all these PEs (Supplementary Figures 1–6).

### Each of the fifteen geroprotective PEs intensifies mitochondrial respiration and alters the pattern of age-related changes in intracellular ROS

**Figure 5:**
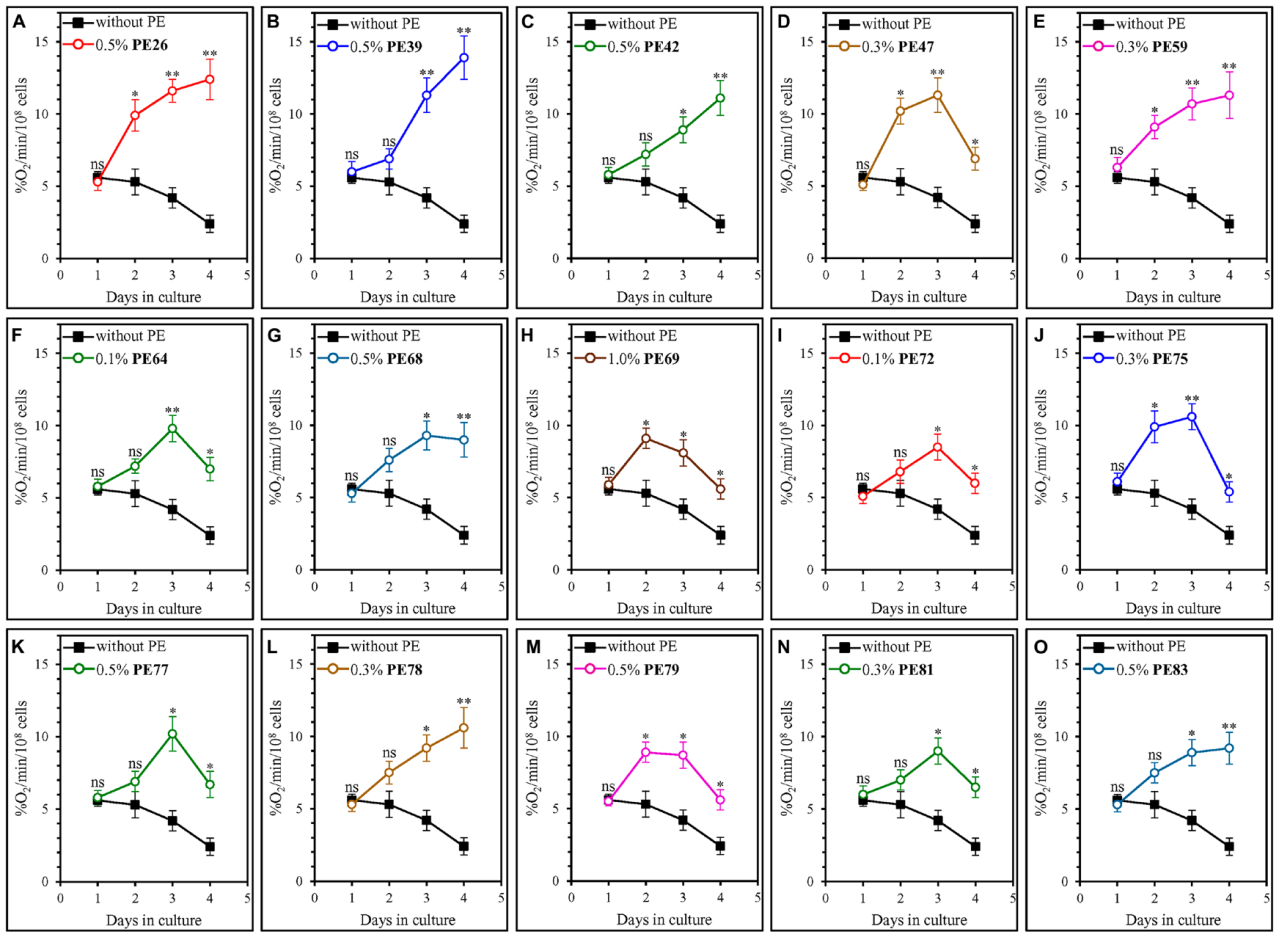
Each of the fifteen geroprotective PEs stimulates mitochondrial respiration in yeast cultured under non-CR conditions. WT cells were cultured in the synthetic minimal YNB medium initially containing 2% (w/v) glucose, in the presence of a PE or its absence. The following PEs were added to the culture: 0.5% (w/v) PE26 (**A**), 0.5% (w/v) PE39 (**B**), 0.5% (w/v) PE42 (**C**), 0.3% (w/v) PE47 (**D**), 0.3% (w/v) PE59 (**E**), 0.1% (w/v) PE64 (**F**), 0.5% (w/v) PE68 (**G**), 1.0% (w/v) PE69 (**H**), 0.1% (w/v) PE72 (**I**), 0.3% (w/v) PE75 (**J**), 0.5% (w/v) PE77 (**K**), 0.3% (w/v) PE78 (**L**), 0.5% (w/v) PE79 (**M**), 0.3% (w/v) PE81 (**N**) or 0.5% (w/v) PE83 (**O**). In the cultures supplemented with a PE, ethanol was used as a vehicle at a final concentration of 2.5% (v/v). In the same experiment, WT cells were also subjected to ethanol-mock treatment by being cultured in the synthetic minimal YNB medium initially containing 2% (w/v) glucose and 2.5% (v/v) ethanol. Oxygen uptake by live yeast cells was measured using polarography, as described in Materials and Methods. Age-related changes in the rate of mitochondrial oxygen consumption are shown. Data are presented as means ± SEM (*n* = 3; ^*^ *p* < 0.05, ^**^ *p* < 0.01, *ns*, not significant; the *p* values for comparing the means of two in groups were calculated using an unpaired two-tailed *t* test as described in Materials and Methods).

A distinct set of cellular processes is known to define the rate of yeast chronological aging [1, 4, 64–72]. These processes include coupled mitochondrial respiration [1, 4, 64, 69, 73–77]. We investigated how each of the fifteen geroprotective PEs influences an age-related chronology of changes in coupled mitochondrial respiration, which we measured as the rate of oxygen consumption by yeast cells. We found that each of these PEs causes a statistically significant increase in the rate of mitochondrial respiration on days 3 and 4 of culturing in the YNB medium initially containing 2% (w/v) glucose (Figure 5). On these days of culturing in the YNB medium with 2% (w/v) glucose, yeast cells are known to enter and proceed through a stationary (ST) phase of culturing [33].

We found that the fifteen geroprotective PEs belong to two different groups regarding their effects on the age-related dynamics of changes in coupled mitochondrial respiration under non-CR conditions. The first group of these PEs includes PE47, PE64, PE69, PE72, PE75, PE77, PE79 and PE81. Although all these geroprotective PEs allowed the yeast to maintain the rates of mitochondrial respiration significantly exceeding those in yeast subjected to ethanol-mock treatment, none of them prevented an age-related decline in mitochondrial respiration during the ST phase of culturing (Figure 5D, 5F, 5H–5K, 5M and 5N). Of note, all geroprotective PEs from the first group were able to extend yeast CLS only under non-CR conditions on 2% (w/v) glucose (Figures 1D, 1F, 1H, 2A, 2B, 2C, 2E and 2F) but not under CR conditions on 0.5% (w/v) glucose (Supplementary Figures 10D, 10F, 10H, 11A, 11B, 11C, 11E and 11F). The second group of geroprotective PEs includes PE26, PE39, PE42, PE59, PE68, PE78 and PE83. These PEs increased the rate of mitochondrial respiration and sustained it high in ST-phase cultures that were recovered on day 4 (Figure 5A, 5B, 5C, 5E, 5G, 5L and 5O). Noteworthy, all geroprotective PEs from the second group were able to extend yeast CLS under both non-CR conditions on 2% (w/v) glucose (Figures 1A–1C, 1E, 1G, 2D and 2G) and CR conditions on 0.5% (w/v) glucose (Supplementary Figures 10A–10C, 10E, 10G, 11D and 11G). As we hypothesized earlier in the text, it is conceivable that the geroprotective PEs from the first group could target only CR-regulated cellular processes (including mitochondrial respiration), whereas the geroprotective PEs from the second group could target both CR-regulated and housekeeping (i.e., not regulated by CR) cellular processes (including mitochondrial respiration).

Several ROS are known to be the primary by-products of coupled mitochondrial respiration [78–80]. These ROS of mitochondrial origin are known for their essential roles in defining the rate of aging in organisms across species, including *S. cerevisiae* [69, 79–87].

**Figure 6:**
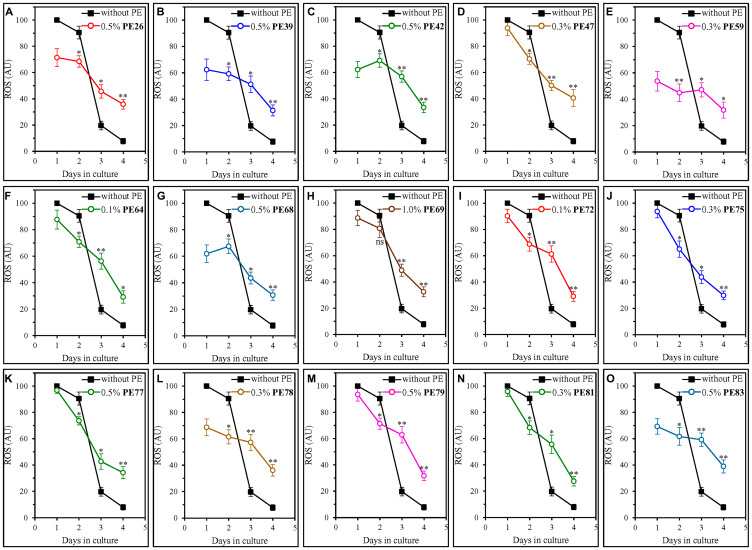
Each of the fifteen geroprotective PEs alters the age-related chronology of changes in intracellular ROS in yeast cultured under non-CR conditions. WT cells were cultured in the synthetic minimal YNB medium initially containing 2% (w/v) glucose, in the presence of a PE or its absence. The following PEs were added to the culture: 0.5% (w/v) PE26 (**A**), 0.5% (w/v) PE39 (**B**), 0.5% (w/v) PE42 (**C**), 0.3% (w/v) PE47 (**D**), 0.3% (w/v) PE59 (**E**), 0.1% (w/v) PE64 (**F**), 0.5% (w/v) PE68 (**G**), 1.0% (w/v) PE69 (**H**), 0.1% (w/v) PE72 (**I**), 0.3% (w/v) PE75 (**J**), 0.5% (w/v) PE77 (**K**), 0.3% (w/v) PE78 (**L**), 0.5% (w/v) PE79 (**M**), 0.3% (w/v) PE81 (**N**) or 0.5% (w/v) PE83 (**O**). In the cultures supplemented with a PE, ethanol was used as a vehicle at a final concentration of 2.5% (v/v). In the same experiment, WT cells were also subjected to ethanol-mock treatment by being cultured in the synthetic minimal YNB medium initially containing 2% (w/v) glucose and 2.5% (v/v) ethanol. The intracellular concentrations of ROS were measured in live yeast by fluorescence microscopy of dihydrorhodamine 123 staining, as described in Materials and Methods. Age-related changes in the intracellular concentration of ROS are shown. Data are presented as means ± SEM (*n* = 3; ^*^ *p* < 0.05, ^**^ *p* < 0.01, *ns*, not significant; the *p* values for comparing the means of two in groups were calculated using an unpaired two-tailed *t* test as described in Materials and Methods).

We found that all fifteen geroprotective PEs alter the age-related dynamics of changes in intracellular ROS (Figure 6). Each of these PEs slowed an age-related decline in intracellular ROS on days 3 and 4 of culturing, thus enabling a moderate but statistically significant rise in intracellular ROS during the ST phase (Figure 6). During the post-diauxic (PD) phase on day 2 of culturing, most of the fifteen geroprotective PEs (other than PE69; Figure 6H) elicited a modest but statistically decline in intracellular ROS (Figure 6).

Noteworthy, as described below, we found that there are two different groups of geroprotective PEs with respect to their effects on intracellular ROS during the logarithmic (L) phase on day 1.

PE47, PE64, PE69, PE72, PE75, PE77, PE79 and PE81 did not elicit a substantial change in intracellular ROS during the L phase of culturing on day 1 (Figure 6D, 6F, 6H, 6I–6K, 6M and 6N). All of them extended yeast CLS only under non-CR conditions on 2% (w/v) glucose (Figures 1D, 1F, 1H, 2A, 2B, 2C, 2E and 2F) but not under CR conditions on 0.5% (w/v) glucose (Supplementary Figures 10D, 10F, 10H, 11A, 11B, 11C, 11E and 11F).

In contrast, PE26, PE39, PE42, PE59, PE68, PE78 and PE83 caused a substantial decline in intracellular ROS during the L phase of culturing on day 1 (Figure 6A–6C, 6E, 6G, 6L and 6O). All these geroprotective PEs stimulated mitochondrial respiration and sustained it high in ST-phase cultures (Figure 5A–5C, 5E, 5G, 5L and 5O). All of them were also capable of prolonging yeast CLS under both non-CR conditions on 2% (w/v) glucose (Figures 1A, 1B, 1C, 1E, 1G, 2D and 2G) and CR conditions on 0.5% (w/v) glucose (Supplementary Figures 10A–10C, 10E, 10G, 11D and 11G).

### Each of the fifteen geroprotective PEs decreases the extent of age-related oxidative damage to cellular proteins, and many of them slow the aging-associated buildup of oxidatively impaired membrane lipids as well as mitochondrial and nuclear DNA

An age-related rise in the intracellular ROS above a toxic threshold has been shown to cause oxidative damage to cellular proteins, lipids and nucleic acids [65, 78–80, 84–95]. The aging-associated accumulation of these oxidized macromolecules is one of the essential contributors to the aging process in yeast and other organisms [65, 78–80, 84–95].

Each of the fifteen geroprotective PEs perturbed the age-related chronology of changes in intracellular ROS (see above). Therefore, we investigated whether each of them also influences the aging-associated accumulation of oxidatively impaired proteins, lipids and DNA in yeast cells cultured under non-CR conditions on 2% (w/v) glucose.

**Figure 7:**
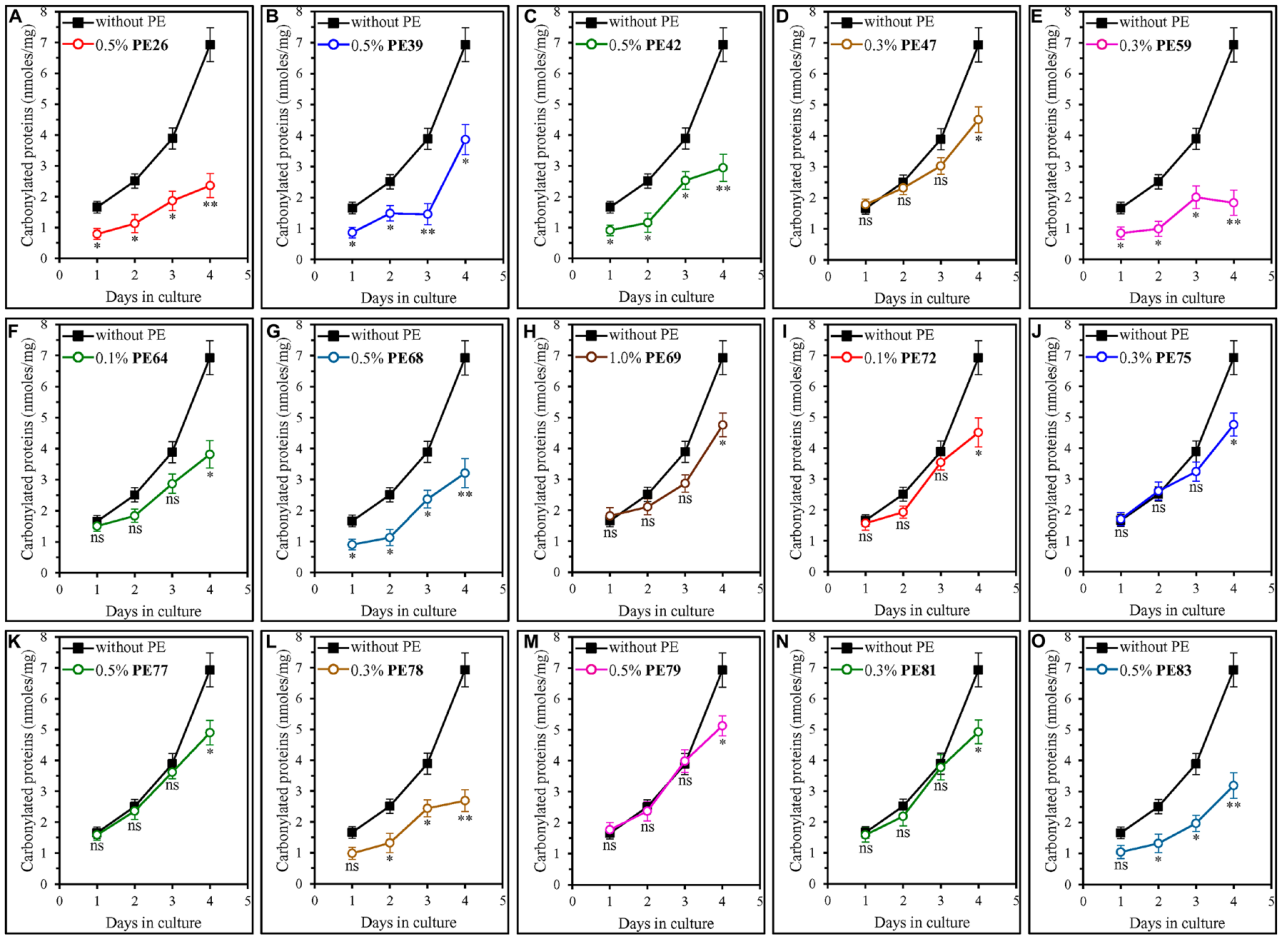
Each of the fifteen geroprotective PEs decreases the extent of age-related oxidative damage to cellular proteins in yeast cultured under non-CR conditions. WT cells were cultured in the synthetic minimal YNB medium initially containing 2% (w/v) glucose, in the presence of a PE or its absence. The following PEs were added to the culture: 0.5% (w/v) PE26 (**A**), 0.5% (w/v) PE39 (**B**), 0.5% (w/v) PE42 (**C**), 0.3% (w/v) PE47 (**D**), 0.3% (w/v) PE59 (**E**), 0.1% (w/v) PE64 (**F**), 0.5% (w/v) PE68 (**G**), 1.0% (w/v) PE69 (**H**), 0.1% (w/v) PE72 (**I**), 0.3% (w/v) PE75 (**J**), 0.5% (w/v) PE77 (**K**), 0.3% (w/v) PE78 (**L**), 0.5% (w/v) PE79 (**M**), 0.3% (w/v) PE81 (**N**) or 0.5% (w/v) PE83 (**O**). In the cultures supplemented with a PE, ethanol was used as a vehicle at a final concentration of 2.5% (v/v). In the same experiment, WT cells were also subjected to ethanol-mock treatment by being cultured in the synthetic minimal YNB medium initially containing 2% (w/v) glucose and 2.5% (v/v) ethanol. The concentrations of oxidatively damaged (carbonylated) proteins were measured as described in Materials and Methods. Age-related changes in the intracellular concentration (nmoles/mg protein) of carbonylated proteins are shown. Data are presented as means ± SEM (*n* = 3; ^*^ *p* < 0.05, ^**^ *p* < 0.01, *ns*, not significant; the *p* values for comparing the means of two in groups were calculated using an unpaired two-tailed *t* test as described in Materials and Methods).

We found that all fifteen geroprotective PEs elicit a statistically significant decline in the abundance of oxidatively damaged (carbonylated) cellular proteins in ST-phase cultures recovered on day 4 (Figure 7).

We noticed that these geroprotective PEs belong to two different groups regarding their effects on the extent of protein carbonylation in yeast cells taken on day 1, 2 or 3 of culturing.

The first group of these PEs includes PE47, PE64, PE69, PE72, PE75, PE77, PE79 and PE81, all of which did not cause a statistically significant decline in the abundance of oxidatively damaged proteins within yeast cells recovered on day 1, 2 or 3 of culturing (Figure 7D, 7F, 7H–7K, 7M and 7N). All geroprotective PEs from the first group extended yeast CLS only under non-CR conditions on 2% (w/v) glucose (Figures 1D, 1F, 1H, 2A, 2B, 2C, 2E and 2F) but not under CR conditions on 0.5% (w/v) glucose (Supplementary Figures 10D, 10F, 10H, 11A, 11B, 11C, 11E and 11F).

The second group of geroprotective PEs includes PE26, PE39, PE42, PE59, PE68, PE78 and PE83, all of which substantially lowered the abundance of oxidatively damaged proteins in yeast recovered on day 1, 2 or 3 of culturing (Figure 7A–7C, 7E, 7G, 7L and 7O). Only for PE78 and PE83 such effects on protein carbonylation were not statistically significant in yeast taken on day 1 of culturing (Figure 7L and 7O). All geroprotective PEs from the second group increased yeast CLS under both non-CR conditions on 2% (w/v) glucose (Figures 1A–1C, 1E, 1G, 2D and 2G) and CR conditions on 0.5% (w/v) glucose (Supplementary Figures 10A, 10B, 10C, 10E, 10G, 11D and 11G).

**Figure 8:**
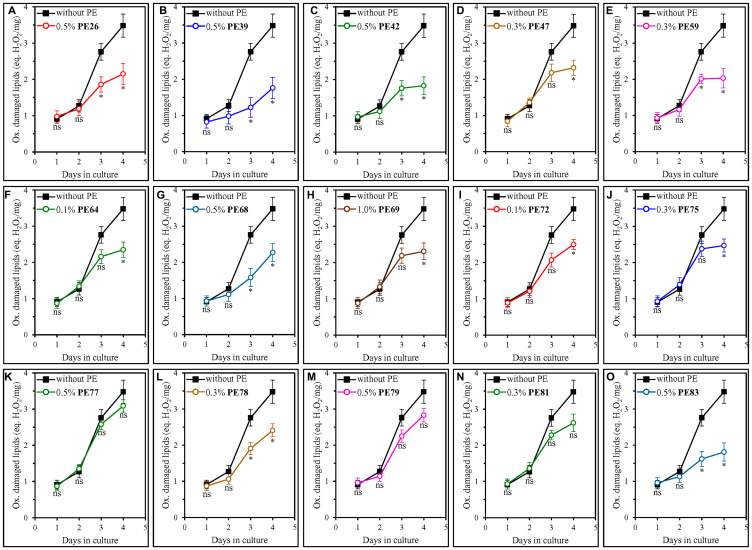
Many of the fifteen geroprotective PEs slow the aging-associated buildup of oxidatively impaired membrane lipids in yeast cultured under non-CR conditions. WT cells were cultured in the synthetic minimal YNB medium initially containing 2% (w/v) glucose, in the presence of a PE or its absence. The following PEs were added to the culture: 0.5% (w/v) PE26 (**A**), 0.5% (w/v) PE39 (**B**), 0.5% (w/v) PE42 (**C**), 0.3% (w/v) PE47 (**D**), 0.3% (w/v) PE59 (**E**), 0.1% (w/v) PE64 (**F**), 0.5% (w/v) PE68 (**G**), 1.0% (w/v) PE69 (**H**), 0.1% (w/v) PE72 (**I**), 0.3% (w/v) PE75 (**J**), 0.5% (w/v) PE77 (**K**), 0.3% (w/v) PE78 (**L**), 0.5% (w/v) PE79 (**M**), 0.3% (w/v) PE81 (**N**) or 0.5% (w/v) PE83 (**O**). In the cultures supplemented with a PE, ethanol was used as a vehicle at a final concentration of 2.5% (v/v). In the same experiment, WT cells were also subjected to ethanol-mock treatment by being cultured in the synthetic minimal YNB medium initially containing 2% (w/v) glucose and 2.5% (v/v) ethanol. The concentrations of oxidatively damaged membrane lipids were measured as described in Materials and Methods. Age-related changes in the intracellular concentration (equivalents of nmoles H_2_O_2_/mg protein) of oxidatively damaged membrane lipids are shown. Data are presented as means ± SEM (*n* = 3; ^*^ *p* < 0.05, *ns*, not significant; the *p* values for comparing the means of two in groups were calculated using an unpaired two-tailed *t* test as described in Materials and Methods).

Our analysis of how each of the fifteen geroprotective PEs influences the extent of oxidative damage to membrane lipids revealed that PE26, PE39, PE42, PE47, PE59, PE64, PE68, PE69, PE72, PE75, PE78 and PE83 statistically significantly decrease it in ST-phase cultures recovered on day 4 (Figure 8A–8J, 8L and 8O). For PE77, PE79 and PE81, a decline in the abundance of oxidatively impaired membrane lipids in yeast cells taken on day 4 of culturing was noticeable but not statistically significant (Figure 8K, 8M and 8N). We also found that only those of the fifteen geroprotective PEs that extend yeast CLS under both non-CR and CR conditions significantly lower the abundance of oxidized membrane lipids even in yeast recovered on day 3 of culturing on 2% (w/v) glucose (Figure 8A–8C, 8E, 8G, 8L and 8O for PE26, PE39, PE42, PE59, PE68, PE78 and PE83). In contrast, a decline in the abundance of oxidatively damaged membrane lipids on day 3 of culturing on 2% (w/v) glucose was noticeable but not statistically significant for any of the geroprotective PEs that increased yeast CLS only under non-CR conditions (Figure 8D, 8F, 8H–8K, 8M and 8N for PE47, PE64, PE69, PE72, PE75, PE77, PE79 and PE81).

**Figure 9:**
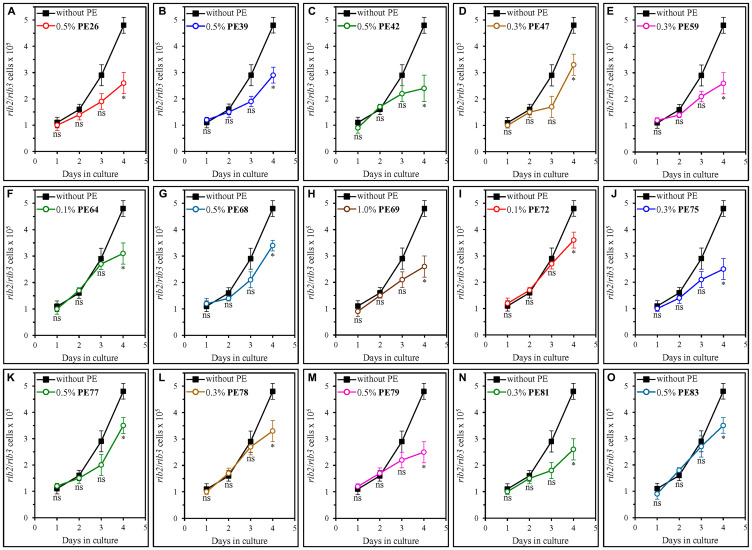
Each of the fifteen geroprotective PEs decreases the frequencies of rib2 and rib3 mutations in mitochondrial DNA (mtDNA) of yeast cultured under non-CR conditions. WT cells were cultured in the synthetic minimal YNB medium initially containing 2% (w/v) glucose, in the presence of a PE or its absence. The following PEs were added to the culture: 0.5% (w/v) PE26 (**A**), 0.5% (w/v) PE39 (**B**), 0.5% (w/v) PE42 (**C**), 0.3% (w/v) PE47 (**D**), 0.3% (w/v) PE59 (**E**), 0.1% (w/v) PE64 (**F**), 0.5% (w/v) PE68 (**G**), 1.0% (w/v) PE69 (**H**), 0.1% (w/v) PE72 (**I**), 0.3% (w/v) PE75 (**J**), 0.5% (w/v) PE77 (**K**), 0.3% (w/v) PE78 (**L**), 0.5% (w/v) PE79 (**M**), 0.3% (w/v) PE81 (**N**) or 0.5% (w/v) PE83 (**O**). In the cultures supplemented with a PE, ethanol was used as a vehicle at a final concentration of 2.5% (v/v). In the same experiment, WT cells were also subjected to ethanol-mock treatment by being cultured in the synthetic minimal YNB medium initially containing 2% (w/v) glucose and 2.5% (v/v) ethanol. The incidences of spontaneous point mutations in the *RIB2* and *RIB3* genes of mtDNA were measured as described in Materials and Methods. Age-related changes in the frequencies of these mtDNA mutations are shown. Data are presented as means ± SEM (*n* = 3; ^*^ *p* < 0.05, *ns*, not significant; the *p* values for comparing the means of two in groups were calculated using an unpaired two-tailed *t* test as described in Materials and Methods).

**Figure 10:**
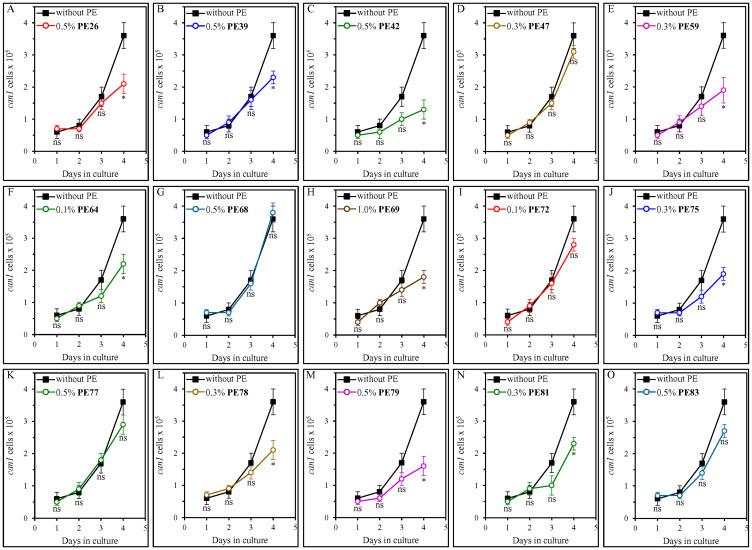
PE26, PE39, PE42, PE59, PE64, PE69, PE75, PE78, PE79 and PE81 (but not PE47, PE68, PE72, PE77 or PE83) cause a statistically significant decline in the frequencies of can1 mutations in nuclear DNA (nDNA) of yeast cultured under non-CR conditions. WT cells were cultured in the synthetic minimal YNB medium initially containing 2% (w/v) glucose, in the presence of a PE or its absence. The following PEs were added to the culture: 0.5% (w/v) PE26 (**A**), 0.5% (w/v) PE39 (**B**), 0.5% (w/v) PE42 (**C**), 0.3% (w/v) PE47 (**D**), 0.3% (w/v) PE59 (**E**), 0.1% (w/v) PE64 (**F**), 0.5% (w/v) PE68 (**G**), 1.0% (w/v) PE69 (**H**), 0.1% (w/v) PE72 (**I**), 0.3% (w/v) PE75 (**J**), 0.5% (w/v) PE77 (**K**), 0.3% (w/v) PE78 (**L**), 0.5% (w/v) PE79 (**M**), 0.3% (w/v) PE81 (**N**) or 0.5% (w/v) PE83 (**O**). In the cultures supplemented with a PE, ethanol was used as a vehicle at a final concentration of 2.5% (v/v). In the same experiment, WT cells were also subjected to ethanol-mock treatment by being cultured in the synthetic minimal YNB medium initially containing 2% (w/v) glucose and 2.5% (v/v) ethanol. The incidences of spontaneous point mutations in the *CAN1* gene of nDNA were measured as described in Materials and Methods. Age-related changes in the frequencies of these nDNA mutations are shown. Data are presented as means ± SEM (*n* = 3; ^*^ *p* < 0.05, *ns*, not significant; the *p* values for comparing the means of two in groups were calculated using an unpaired two-tailed *t* test as described in Materials and Methods).

We also examined how each of the fifteen geroprotective PEs influences the extent of oxidative damage to mitochondrial DNA (mtDNA) and nuclear DNA (nDNA). The oxidative damage to each of these two types of DNA molecules is known to cause an aging-associated buildup of mutations in mtDNA and nDNA [96–100]. Therefore, we investigated the effect of each of the fifteen geroprotective PEs on the frequencies of spontaneous point mutations in the *RIB2* and *RIB3* genes of mtDNA [64, 101] as well as the frequencies of spontaneous point mutations in the *CAN1* gene of nDNA [64, 101]. We found that all fifteen geroprotective PEs statistically significantly decrease the incidences of *rib2* and *rib3* mutations in mtDNA of yeast recovered from the ST phase on day 4 but not on any other day of culturing (Figure 9). Furthermore, PE26, PE39, PE42, PE59, PE64, PE69, PE75, PE78, PE79 and PE81 caused a statistically significant decline in the frequencies of *can1* mutations in nDNA of yeast cells that were taken from the ST phase on day 4 of culturing only (Figure 10A–10C, 10E, 10F, 10H, 10J and 10L–10N). In contrast, neither PE47, PE68, PE72, PE77 nor PE83 elicited a significant change in the incidences of these mutations in nDNA of yeast recovered on any day of culturing (Figure 10D, 10G, 10I, 10K and 10O, respectively).

### Each of the fifteen geroprotective PEs increases cell resistance to long-term oxidative and thermal stresses

Genetic, dietary and chemical interventions that decrease cell susceptibility to chronic (long-term) oxidative and/or thermal stresses have been shown to decelerate the aging process and extend longevity in yeast and other organisms across species [1, 4, 60–64, 78, 102–106]. Therefore, we investigated the effect of each of the fifteen geroprotective PEs on the susceptibility of chronologically aging yeast cells to these two types of chronic stresses.

To examine aging-associated changes in cell susceptibility to these long-term stresses, we recovered aliquots of yeast cells on days 1, 2, 3 and 4 of culturing under non-CR conditions in liquid YNB medium with 2% (w/v) glucose. To assess cell susceptibility to chronic oxidative stress, we spotted serial dilutions of these cell aliquots on solid YEP medium with 2% (w/v) glucose and 5 mM hydrogen peroxide and incubated them for 3 days. To assess cell susceptibility to chronic thermal stress, we spotted serial dilutions of these cell aliquots on solid YEP medium with 2% (w/v) glucose, incubated at 60°C for 60 min, transferred the plates to 30°C and incubated at this temperature for 3 days.

**Figure 11:**
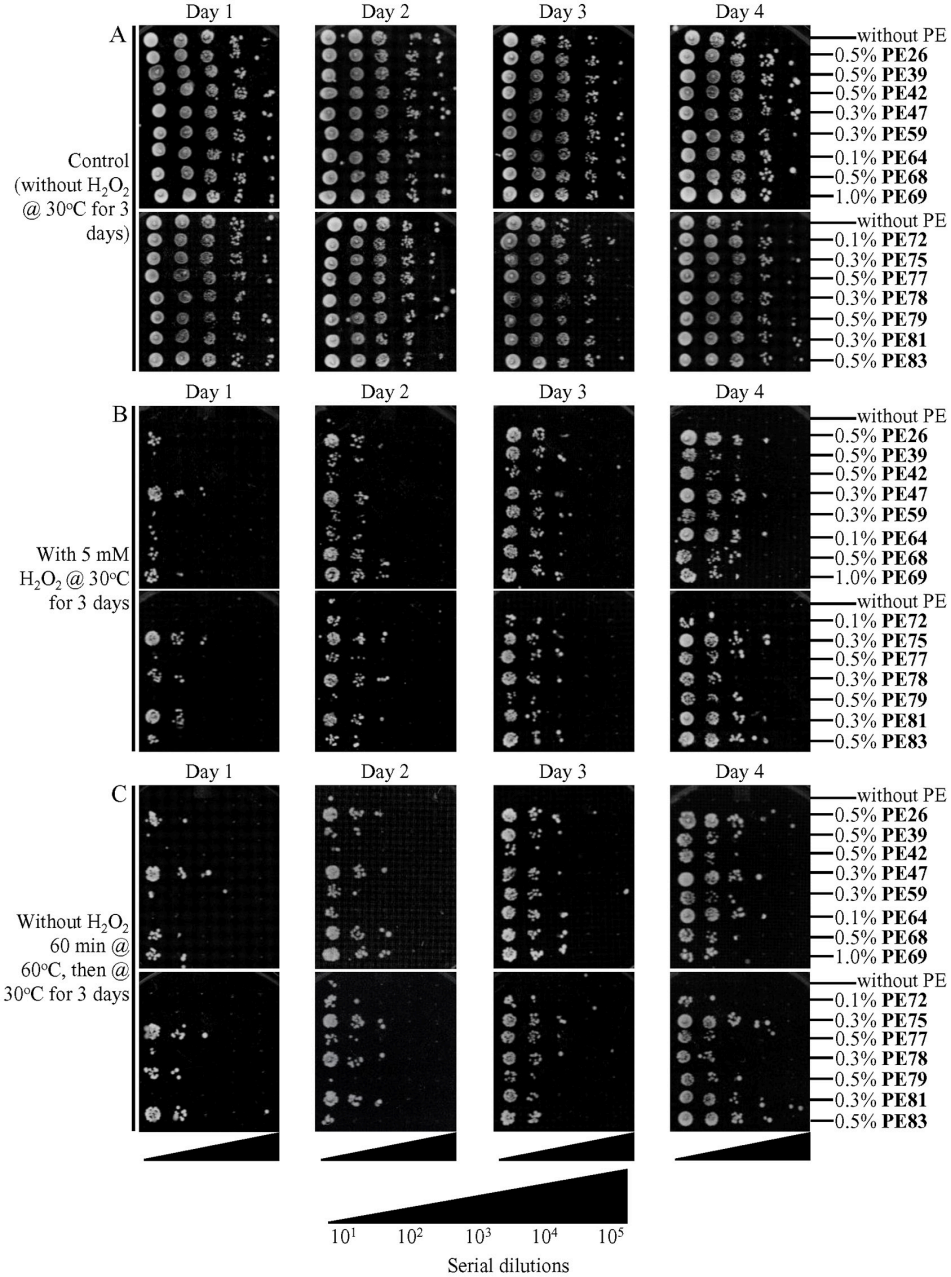
Each of the fifteen geroprotective PEs makes yeast more resistant to chronic (long-term) oxidative and thermal stresses. WT cells were cultured in the synthetic minimal YNB medium initially containing 2% (w/v) glucose, in the presence of a PE or its absence. In the cultures supplemented with a PE, ethanol was used as a vehicle at a final concentration of 2.5% (v/v). In the same experiment, WT cells were also subjected to ethanol-mock treatment by being cultured in the synthetic minimal YNB medium initially containing 2% (w/v) glucose and 2.5% (v/v) ethanol. Spot assays for examining cell resistance to chronic oxidative (**B**) and thermal (**C**) stresses were performed as described in Materials and Methods. (**A**) In control samples, serial 10-fold dilutions of cells recovered on different days of culturing were spotted on plates with solid YEP medium containing 2% (w/v) glucose. All pictures were taken after a 3-d incubation at 30°C. (B) In samples subjected to long-term oxidative stress, serial 10-fold dilutions of cells recovered on different days of culturing were spotted on plates with solid YEP medium containing 2% (w/v) glucose and 5 mM hydrogen peroxide. All pictures were taken after a 3-d incubation at 30°C. (C) In samples subjected to long-term thermal stress, serial 10-fold dilutions of cells recovered on different days of culturing were spotted on plates with solid YEP medium containing 2% (w/v) glucose, incubated at 60°C for 60 min and then transferred to 30°C. All pictures were taken after a 3-d incubation at 30°C.

We found that each of the fifteen geroprotective PEs makes yeast cells more resistant to chronic oxidative and thermal stresses, especially cells in ST-phase cultures recovered on days 3 and 4 (Figure 11A, 11B and 11C, respectively).

## DISCUSSION

We discovered fifteen PEs that extend the longevity of chronologically aging budding yeast. All these PEs originate from plants used in traditional Chinese and other herbal medicines or the Mediterranean and other long-established diets. However, none of these PEs has been previously known for its ability to prolong lifespan in yeast or other organisms.

Our data indicate that each of the fifteen longevity-extending PEs prolongs yeast CLS not because it slows the metabolism of glucose, the only source of carbon and energy added to the growth medium. We also revealed that the longevity-extending ability of each of the fifteen PEs is not caused by its negative effect on the proliferation of yeast cells. Thus, it seems likely that none of these PEs can prolong yeast CLS because it slows the formation and release of harmful products of cell proliferation.

Our study provides evidence that each of the fifteen longevity-extending PEs satisfies all the criteria previously proposed for a CRM. CRMs are chemical interventions that can mimic the CR-like lifespan-increasing and healthspan-improving effects even if calorie supply is not limited [40–45]. Indeed, we uncovered the following. First, each of the fifteen PEs prolongs yeast CLS under non-CR conditions. Second, none of these PEs impairs glucose uptake and metabolism. Third, each of them exhibits CR-like effects on specific aspects of metabolism and physiology; these effects include an increased rate of coupled mitochondrial respiration, an altered chronology of changes in intracellular ROS, and a decline in the oxidative damage to cellular proteins, membrane lipids and mtDNA. Fourth, each of them makes cells more resistant to long-term oxidative and thermal stresses. Of note, PE26, PE39, PE42, PE59, PE68, PE78 and PE83 can prolong yeast CLS even under CR conditions, when all cellular processes that limit longevity under non-CR conditions are likely to be suppressed. Therefore, it seems conceivable that each of these seven PEs may stimulate the longevity-extending cellular processes and/or may suppress the longevity-shortening cellular processes that operate only under CR conditions. Moreover, as we hypothesized above in the text, each of these seven PEs may target both CR-regulated and housekeeping (i. e., not regulated by CR) cellular processes (including cell susceptibility to long-term oxidative and thermal stresses).

Our analyses of the Gompertz mortality rates and dose-response curves have led us to the following two conclusions. First, each of the fifteen PEs prolongs yeast CLS because it is a geroprotective agent that decreases the rate of chronological aging but has no effect on the baseline mortality rate. Second, each of these PEs promotes a hormetic stress response in chronologically aging yeast, as it is evident from a nonlinear and biphasic (i. e., inverted U-shaped or J-shaped) dose-response curve observed for each of them.

In this study, we discovered that the fifteen geroprotective PEs differently affect three groups of cellular processes in chronologically aging yeast, as summarized below.

First, each of the fifteen geroprotective PEs significantly increases the rate of coupled mitochondrial respiration and slows a decline in intracellular ROS (known to be the primary products of mitochondrial respiration) within yeast cells that enter and proceed through the ST phase of culturing.

Second, each of them substantially suppresses oxidative damage to cellular proteins and mtDNA in ST-phase yeast cells that enter day 4 of culturing. We noticed that twelve of these geroprotective PEs also significantly decrease oxidative damage to membrane lipids in ST-phase yeast cells on day 4, whereas PE77, PE79 and PE81 cause a statistically insignificant decline in oxidized membrane lipids within these cells. We also found that ten of these geroprotective PEs significantly reduce oxidative damage to nDNA, while neither PE47, PE68, PE72, PE77 nor PE83 exhibits such effect on nDNA.

Third, each of them significantly decreases cell susceptibility to long-term oxidative and thermal stresses, especially the susceptibility of yeast cells that enter and proceed through the ST phase of culturing.

### Future perspectives

Our goals for the future research of the fifteen geroprotective PEs described here are outlined below.

First, we are interested in investigating and understanding the molecular and cellular mechanisms through which each of these PEs slows yeast chronological aging. We have recently described mechanisms underlying the aging-delaying action of PE21 [36], an extract from the white willow *Salix alba* we discovered in our previous screen for geroprotective PEs [33].

Second, we would like to explore how each of the fifteen geroprotective PEs may coordinate the information flow through a longevity-defining network of signaling pathways and protein kinases operating in budding yeast and other organisms. This network incorporates the pro-aging TORC1, PKA and PKH1/2 pathways as well as the pro-aging serine/threonine-protein kinase Sch9 [1, 4, 8, 34]. This network also integrates the anti-aging SNF1 and ATG pathways as well as the anti-aging serine/threonine-protein kinase Rim15 [1, 4, 8, 34]. Our recent study has revealed that each of the six geroprotective PEs we discovered in the previous screen [33] slows yeast chronological aging through different functional modules of this longevity-defining signaling network [34]. Of note, pairwise mixes of these six geroprotective PEs slow the process of yeast chronological aging in a synergistic or additive manner only if they include the PEs that target different modules of this network [35]. Therefore, we are interested in investigating how different combinations of the fifteen geroprotective PEs described here influence the extent of yeast chronological aging delay. We will be looking for the combinations of geroprotective PEs that exhibit synergistic or additive effects on the extent of yeast chronological aging delay.

Third, the Health Canada government agency defines thirteen of the fifteen geroprotective PEs described here as the ones that are safe for human consumption [107]. The agency recommends using eight of them as health-improving supplements with clinically proven benefits to human health [107]. Among these health-improving PEs are PE26, PE47, PE59, PE64, PE69, PE75, PE77 and PE83 [107]. For each of them, Health Canada provides a detailed description of source material, routes of administration, doses and dosage forms, uses or purposes, durations of use, risk information, cautions and warnings, contraindications, known adverse reactions, non-medicinal ingredients, specifications, references cited and reviewed, examples of appropriate dosage preparations, and frequencies of use [107]. Our ongoing collaborative research aims to investigate which of the eight geroprotective PEs recommended by Health Canada as healthspan-extending dietary additives for humans can increase the replicative lifespan of cultured human fibroblasts or for delaying the onset of aging-associated human diseases. These diseases include arthritis, diabetes, heart disease, kidney disease, liver dysfunction, sarcopenia, stroke, neurodegenerative diseases (including Parkinson’s, Alzheimer’s and Huntington’s diseases), and many forms of cancer [8, 21, 22, 26, 28, 48, 108–123].

## MATERIALS AND METHODS

### Yeast strains, media and growth conditions

The wild-type (WT) strain *Saccharomyces cerevisiae* BY4742 (*MAT*α *his3Δ1 leu2Δ0 lys2Δ0 ura3Δ0*) from Thermo Scientific/Open Biosystems was grown in a synthetic minimal YNB medium (0.67% (w/v) Yeast Nitrogen Base without amino acids from Fisher Scientific; #DF0919-15-3) initially containing 2% (w/v) or 0.5% (w/v) glucose (#D16-10; Fisher Scientific), 20 mg/l *L*-histidine (# H8125; Sigma), 30 mg/l *L*-leucine (#L8912; Sigma), 30 mg/l *L*-lysine (#L5501; Sigma) and 20 mg/l uracil (#U0750; Sigma), with a PE or without it. A stock solution of each PE in ethanol was made on the day of adding this PE to cell cultures. For each PE, the stock solution was added to growth medium with 2% (w/v) or 0.5% (w/v) glucose immediately following cell inoculation into the medium. In a culture supplemented with a PE, ethanol was used as a vehicle at the final concentration of 2.5% (v/v). In the same experiment, yeast cells were also subjected to ethanol-mock treatment by being cultured in growth medium initially containing 2% (w/v) or 0.5% (w/v) glucose and 2.5% (v/v) ethanol. Cells were cultured at 30°C with rotational shaking at 200 rpm in Erlenmeyer flasks at a “flask volume/medium volume” ratio of 5:1.

### Chronological lifespan assay

A sample of cells was taken from a culture at a certain day following cell inoculation and PE addition into the medium. A fraction of the sample was diluted to determine the total number of cells using a hemacytometer. Another fraction of the cell sample was diluted, and serial dilutions of cells were plated in duplicate onto YEP medium (1% (w/v) yeast extract, 2% (w/v) peptone; both from Fisher Scientific; #BP1422-2 and #BP1420-2, respectively) containing 2% (w/v) glucose (#D16-10; Fisher Scientific) as carbon source. After 2 d of incubation at 30°C, the number of colony-forming units (CFU) per plate was counted. The number of CFU was defined as the number of viable cells in a sample. For each culture, the percentage of viable cells was calculated as follows: (number of viable cells per ml/total number of cells per ml) × 100. The percentage of viable cells in the mid-logarithmic growth phase was set at 100%.

### Miscellaneous procedures

The age-specific mortality rate [46, 48], Gompertz slope or mortality rate coefficient (*G*) [46, 47], and mortality rate doubling time (MRDT) [46, 47] were calculated as previously described. The value of the mortality rate was calculated as the number of cells that lost viability (i. e. are unable to form a colony on the surface of a solid nutrient-rich medium) during each time interval divided by the number of viable (i. e. clonogenic) cells at the end of the interval. The natural logarithms of the mortality rate values for each time point were plotted against days of cell culturing. The coefficient *G* of the age-specific mortality rate was calculated as the slope of the Gompertz mortality line, whereas the value of MRDT was calculated as 0.693/*G*. Oxygen consumption assay for monitoring mitochondrial respiration [64], ROS measurement in live yeast [124], fluorescence microscopy [64], quantitative assays for oxidatively damaged proteins and membrane lipids [125], measurements of the frequencies of spontaneous mutations in mitochondrial and nuclear DNA [126], plating assays for the analysis of resistance to oxidative and thermal stresses [126], and glucose concentration measurement assay [124] have been described elsewhere.

### Statistical analysis

Statistical analysis was performed using Microsoft Excel’s Analysis ToolPack-VBA. All data on cell survival are presented as mean ± SEM. The *p* values for comparing the means of two groups using an unpaired two-tailed *t*-test were calculated with the help of the GraphPad Prism 7 statistics software. The logrank test for comparing each pair of survival curves was performed with GraphPad Prism 7. Two survival curves were considered statistically different if the *p* value was less than 0.05.

## SUPPLEMENTARY MATERIALS





## Abbreviations

ATG1: autophagy protein 1
CLS: chronological lifespan
CR: caloric restriction
CRMs: caloric restriction mimetics
PEs: plant extracts
PKA: protein kinase A
PKH1/2: Pkb-activating kinase homolog proteins 1 and 2
Rim15: a regulator of IME2 protein 15
ROS: reactive oxygen species
SNF1: sucrose non-fermenting protein 1
