# Supplementary material for: Discovery of fifteen new geroprotective plant extracts and identification of cellular processes they affect to prolong the chronological lifespan of budding yeast
Source: Oncotarget. 2020 Jun 9;11(23):2182–203. doi: 10.18632/oncotarget.27615 (PMC7289529; doi:10.18632/oncotarget.27615)
Supplement: Supplementary file 1 [file oncotarget-11-2182-s001.pdf]

Discovery of fifteen new geroprotective plant extracts and identification of cellular processes they affect to prolong the chronological lifespan of budding yeast

SUPPLEMENTARY MATERIALS

Supplementary Table 1: Properties of plant extracts (PEs) used to conduct a new screen for PEs that can prolong the longevity of chronologically aging budding yeast. See Supplementary Table 1

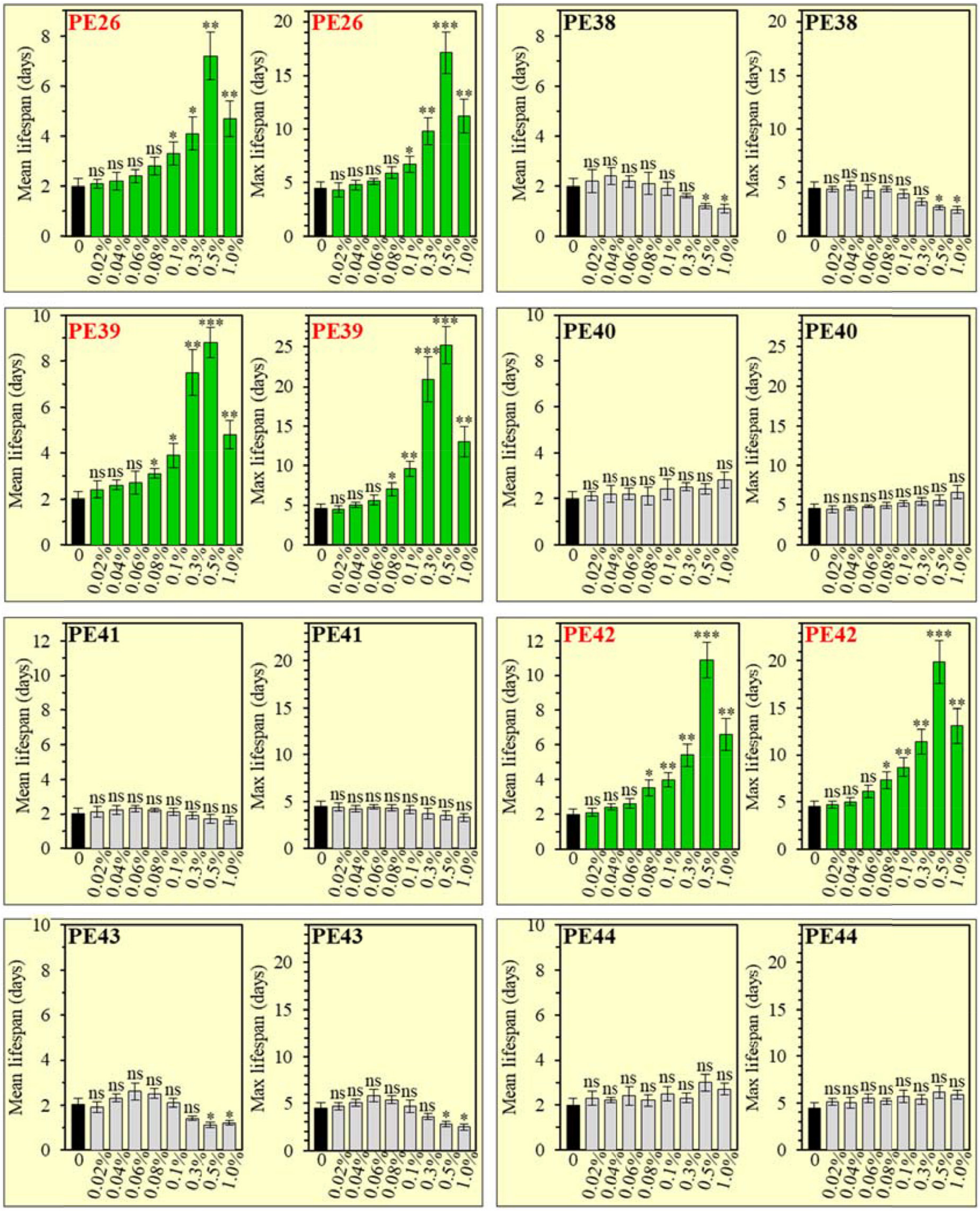

Supplementary Figure 1: PE26, PE39 and PE42, but not PE38, PE40, PE41, PE43 or PE44, increase the mean and maximum CLS of WT yeast cultured under non-CR conditions on 2% (w/v) glucose. WT cells were cultured in the synthetic minimal YNB medium initially containing 2% (w/v) glucose, in the presence of a PE or its absence. The mean and maximum lifespans of chronologically aging WT strain cultured under non-CR conditions without a PE or with a PE added at various concentrations are shown; data are presented as means ± SEM ($n = 6$; $^{*}p < 0.05$, $^{**}p < 0.01$, $^{***}p < 0.001$, ns, not significant; the p values for comparing the means of two groups were calculated using an unpaired two-tailed t test as described in Materials and Methods). Note that PE38 and PE43 can decrease the CLS of WT yeast under non-CR conditions if added at a final concentration of 0.5 (w/v) or 1.0% (w/v).

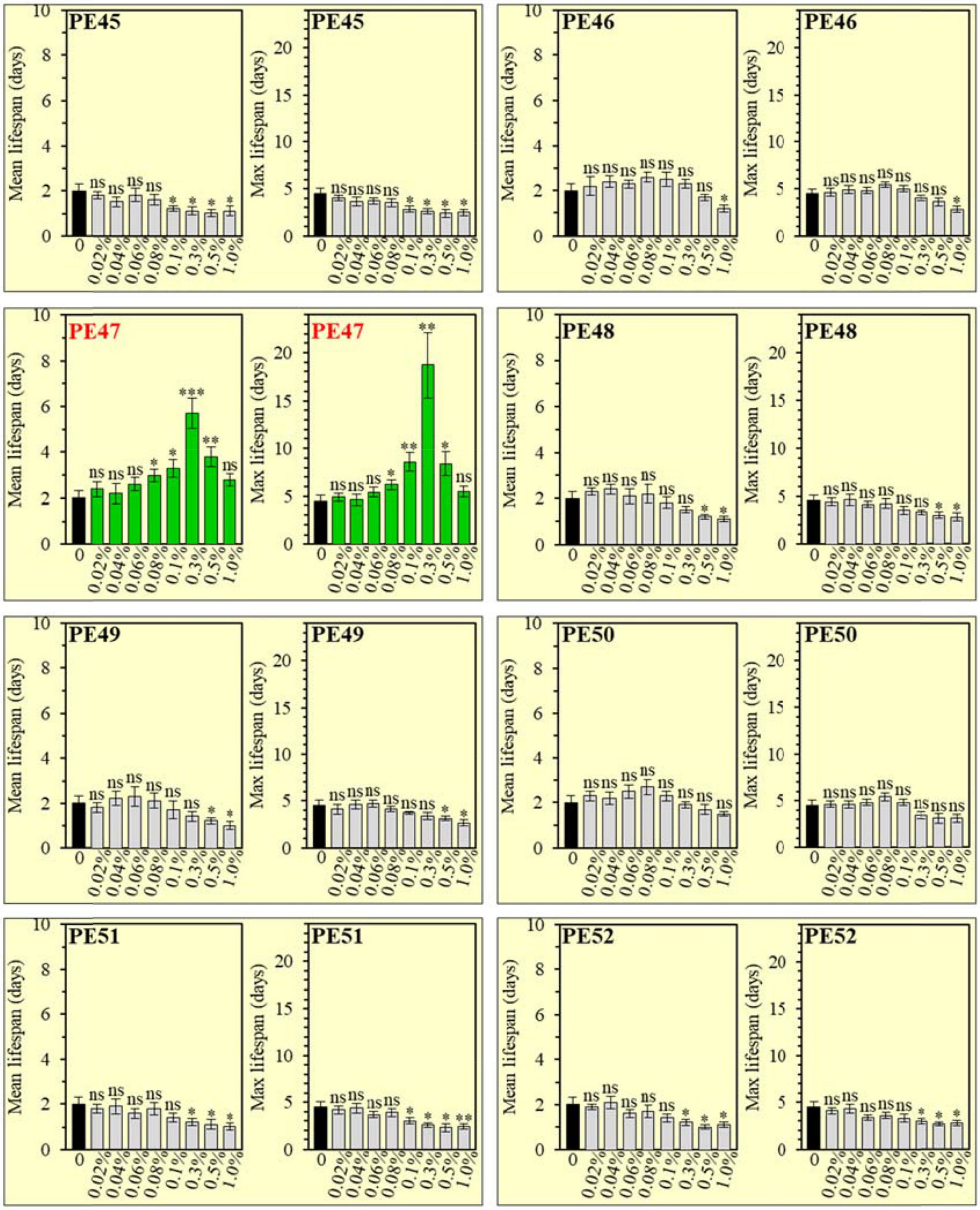

Supplementary Figure 2: PE47, but not PE45, PE46, PE48, PE49, PE50, PE51 or PE52, increases the mean and maximum CLS of WT yeast cultured under non-CR conditions on 2% (w/v) glucose. WT cells were cultured as described in the legend to Supplementary Figure 1. The mean and maximum lifespans of chronologically aging WT strain cultured under non-CR conditions without a PE or with a PE added at various concentrations are shown; data are presented as means ± SEM ($n = 6$; $^{*}p < 0.05$, $^{**}p < 0.01$, $^{***}p < 0.001$, ns, not significant; the p values for comparing the means of two groups were calculated as described in the legend to Supplementary Figure 1). Note that PE45, PE46, PE48, PE49, PE51 and PE52 can decrease the CLS of WT yeast under non-CR conditions if added at a final concentration ranging from 0.1% (w/v) to 1.0% (w/v).

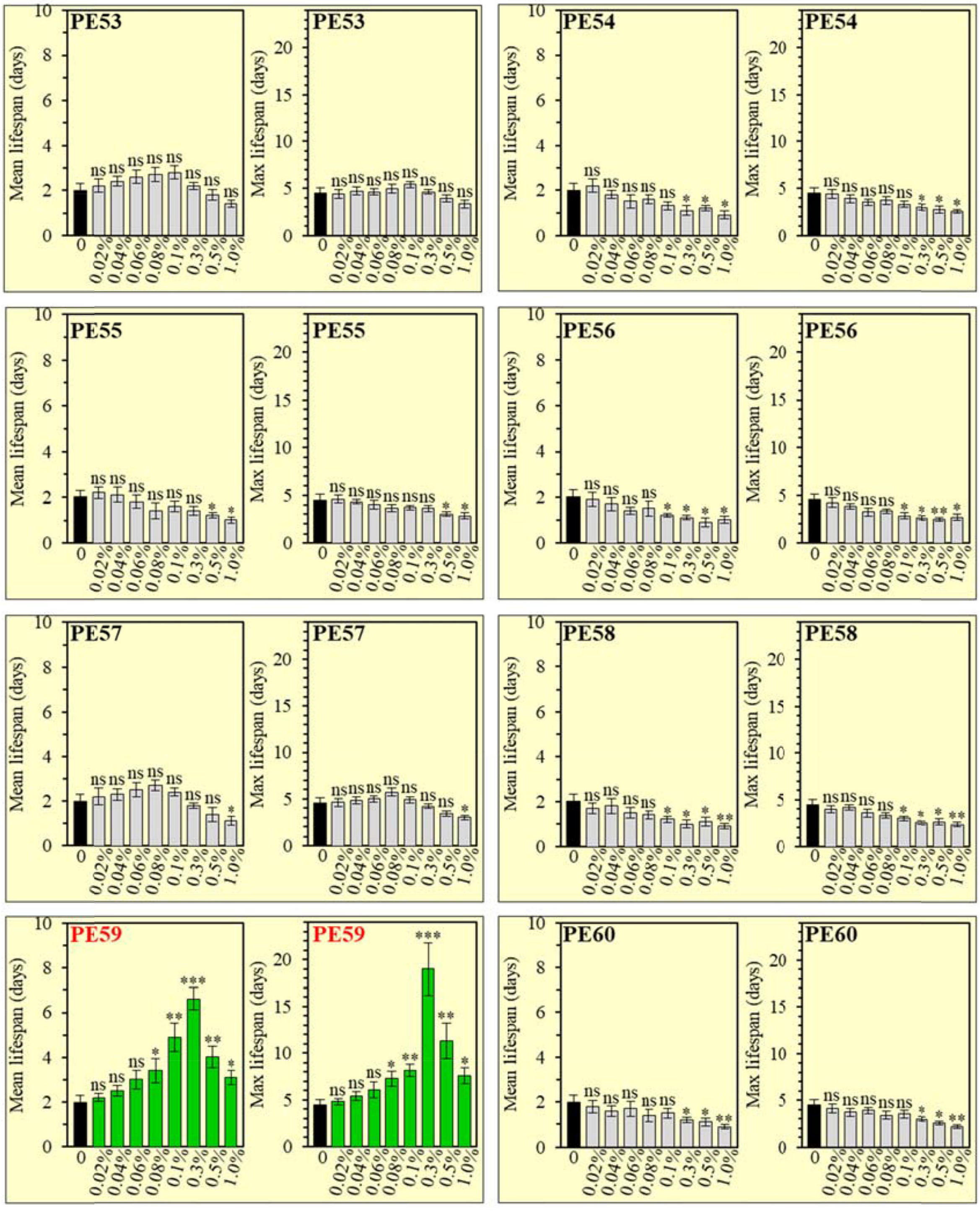

Supplementary Figure 3: PE59, but not PE53, PE54, PE55, PE56, PE57, PE58 or PE60, increases the mean and maximum CLS of WT yeast cultured under non-CR conditions on 2% (w/v) glucose. WT cells were cultured as described in the legend to Supplementary Figure 1. The mean and maximum lifespans of chronologically aging WT strain cultured under non-CR conditions without a PE or with a PE added at various concentrations are shown; data are presented as means ± SEM ($n = 6$; $^{*}p < 0.05$, $^{**}p < 0.01$, $^{***}p < 0.001$, ns, not significant; the p values for comparing the means of two groups were calculated as described in the legend to Supplementary Figure 1). Note that PE54, PE55, PE56, PE57, PE58 and PE60 can decrease the CLS of WT yeast under non-CR conditions if added at a final concentration ranging from 0.1% (w/v) to 1.0% (w/v).

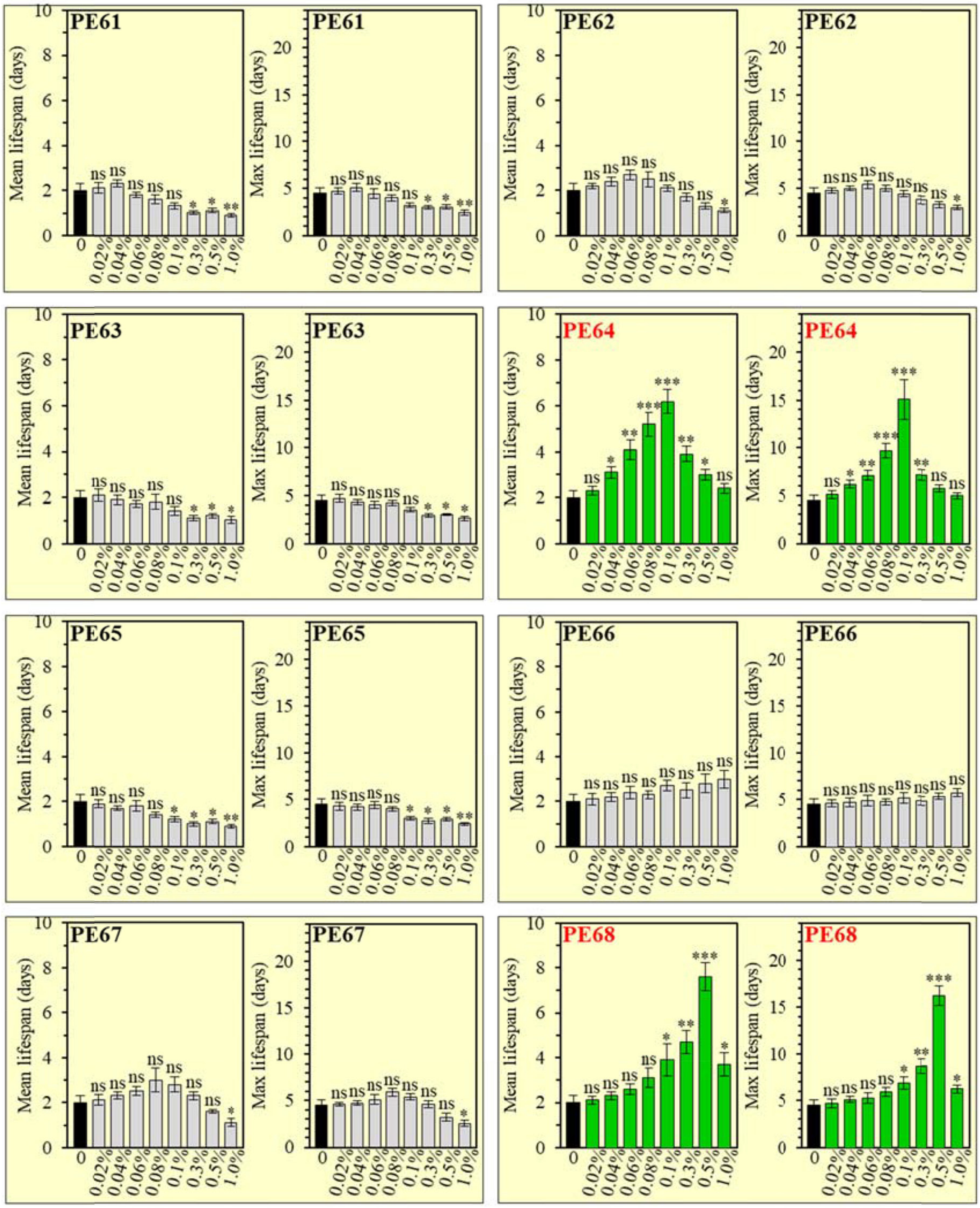

Supplementary Figure 4: PE64 and PE68, but not PE61, PE62, PE63, PE65, PE66 or PE67, increase the mean and maximum CLS of WT yeast cultured under non-CR conditions on 2% (w/v) glucose. WT cells were cultured as described in the legend to Supplementary Figure 1. The mean and maximum lifespans of chronologically aging WT strain cultured under non-CR conditions without a PE or with a PE added at various concentrations are shown; data are presented as means ± SEM ($n = 6$; $^{*}p < 0.05$, $^{**}p < 0.01$, $^{***}p < 0.001$, ns, not significant; the p values for comparing the means of two groups were calculated as described in the legend to Supplementary Figure 1). Note that PE61, PE62, PE63, PE65 and PE67 can decrease the CLS of WT yeast under non-CR conditions if added at a final concentration ranging from 0.1% (w/v) to 1.0% (w/v).

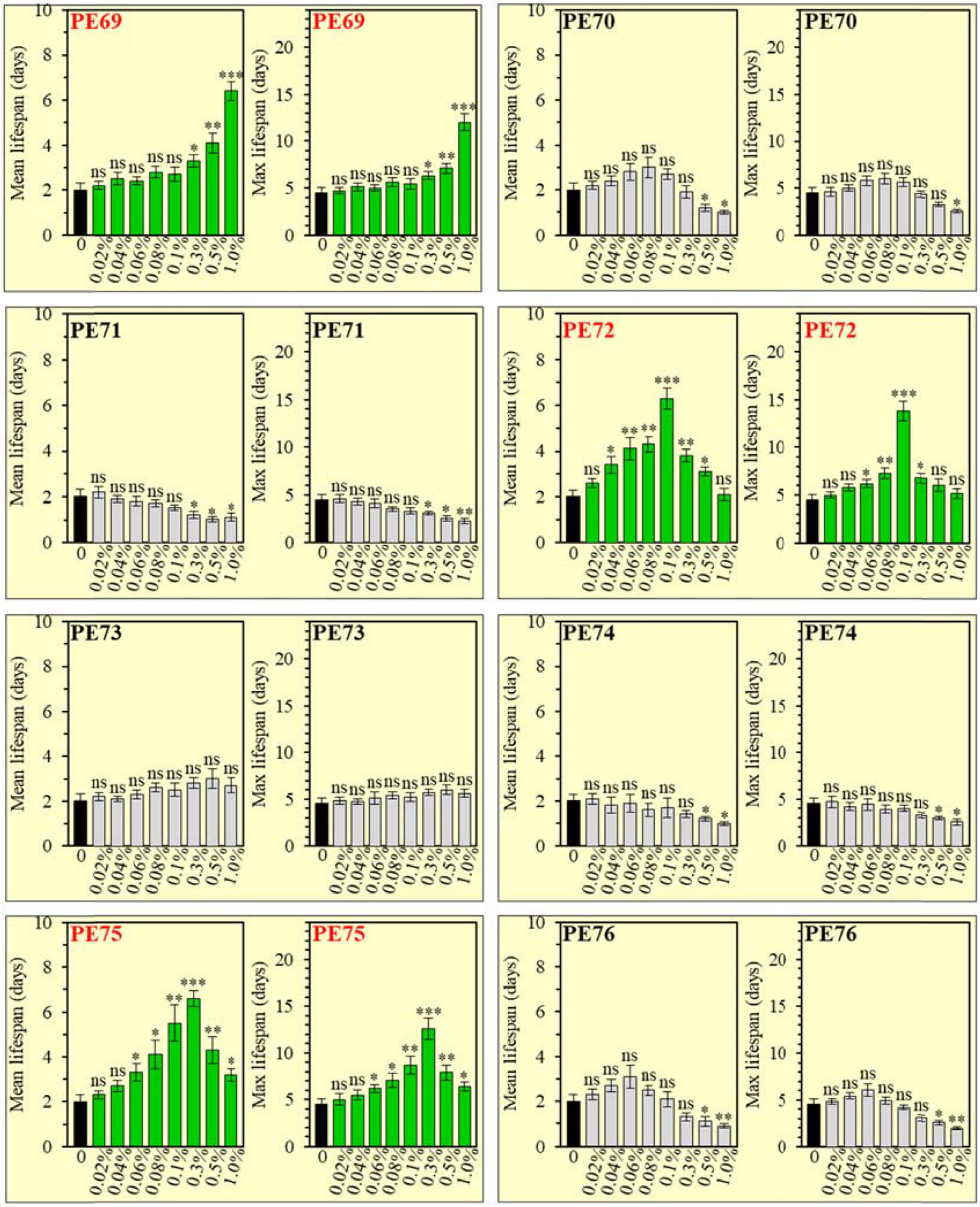

Supplementary Figure 5: PE69, PE72 and PE75, but not PE70, PE71, PE73, PE74 or PE76, increase the mean and maximum CLS of WT yeast cultured under non-CR conditions on 2% (w/v) glucose. WT cells were cultured as described in the legend to Supplementary Figure 1. The mean and maximum lifespans of chronologically aging WT strain cultured under non-CR conditions without a PE or with a PE added at various concentrations are shown; data are presented as means ± SEM (n = 6; $^{*}p < 0.05$, $^{**}p < 0.01$, $^{***}p < 0.001$, ns, not significant; the p values for comparing the means of two groups were calculated as described in the legend to Supplementary Figure 1). Note that PE70, PE71, PE74 and PE76 can decrease the CLS of WT yeast under non-CR conditions if added at a final concentration ranging from 0.3% (w/v) to 1.0% (w/v).

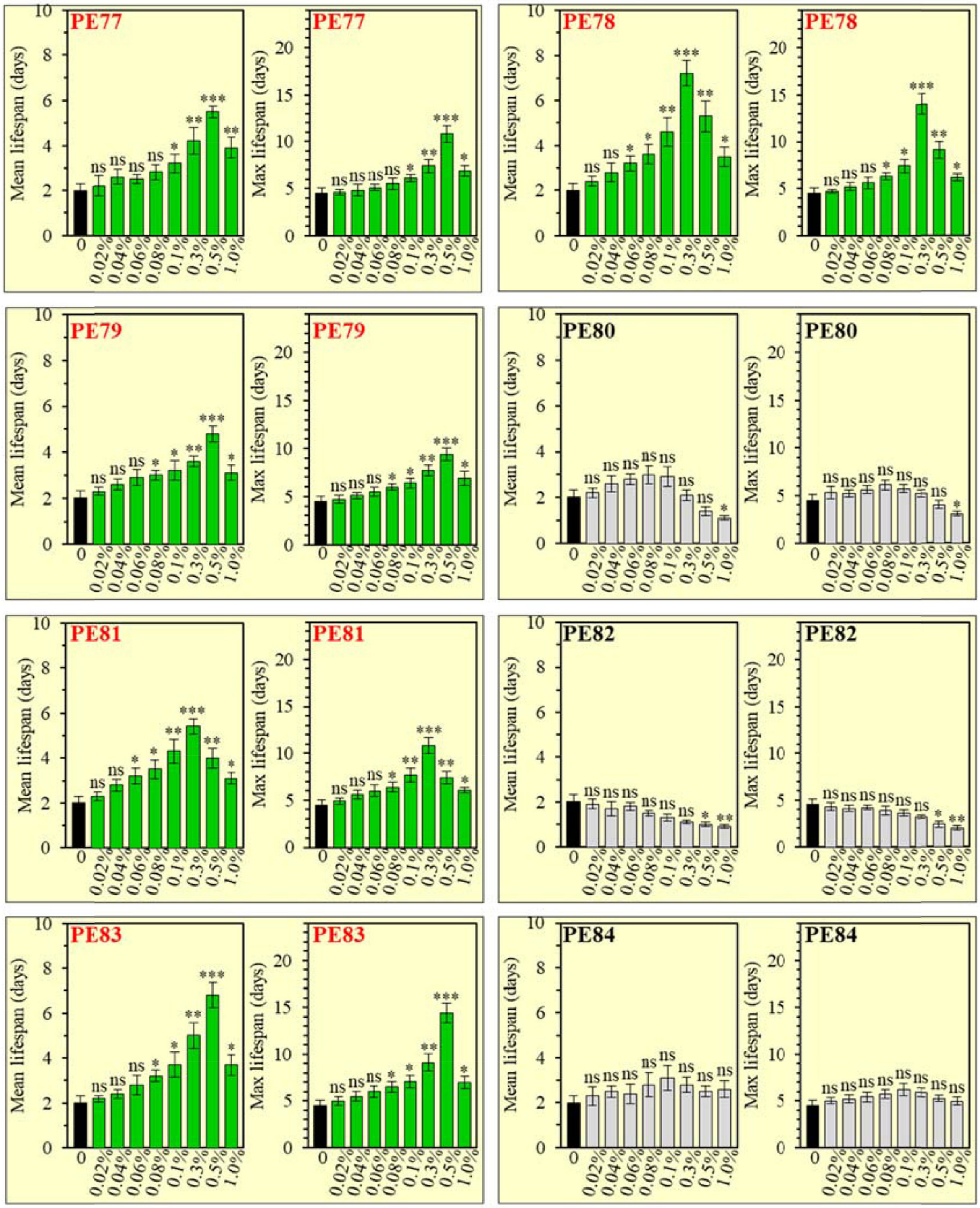

Supplementary Figure 6: PE77, PE78, PE79, PE81 and PE83, but not PE80, PE82 or PE84, increase the mean and maximum CLS of WT yeast cultured under non-CR conditions on 2% (w/v) glucose. WT cells were cultured as described in the legend to Supplementary Figure 1. The mean and maximum lifespans of chronologically aging WT strain cultured under non-CR conditions without a PE or with a PE added at various concentrations are shown; data are presented as means ± SEM ($n = 6$; $^{*}p < 0.05$, $^{**}p < 0.01$, $^{***}p < 0.001$, ns, not significant; the *p* values for comparing the means of two groups were calculated as described in the legend to Supplementary Figure 1). Note that PE80 and PE82 can decrease the CLS of WT yeast under non-CR conditions if added at a final concentration of 0.5% (w/v) or 1.0% (w/v).

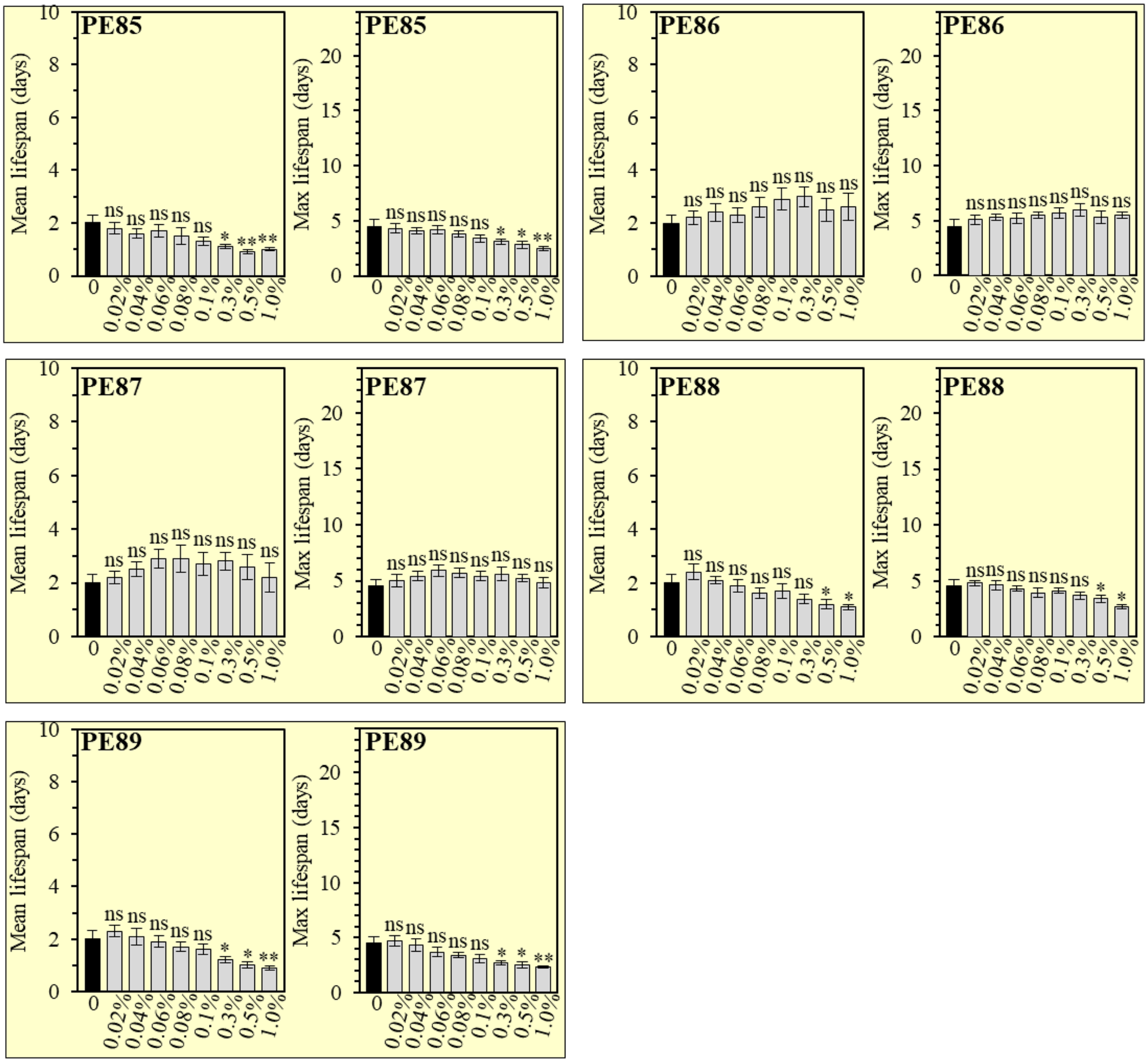

Supplementary Figure 7: Neither PE85, PE86, PE87, PE88 nor PE89 can increase the mean or maximum CLS of WT yeast cultured under non-CR conditions on 2% (w/v) glucose. WT cells were cultured as described in the legend to Supplementary Figure 1. The mean and maximum lifespans of chronologically aging WT strain cultured under non-CR conditions without a PE or with a PE added at various concentrations are shown; data are presented as means ± SEM (n = 6; $^{*}p < 0.05$, $^{**}p < 0.01$, ns, not significant; the p values for comparing the means of two groups were calculated as described in the legend to Supplementary Figure 1). Note that PE85, PE88 and PE89 can decrease the CLS of WT yeast under non-CR conditions if added at a final concentration ranging from 0.3% (w/v) to 1.0% (w/v).

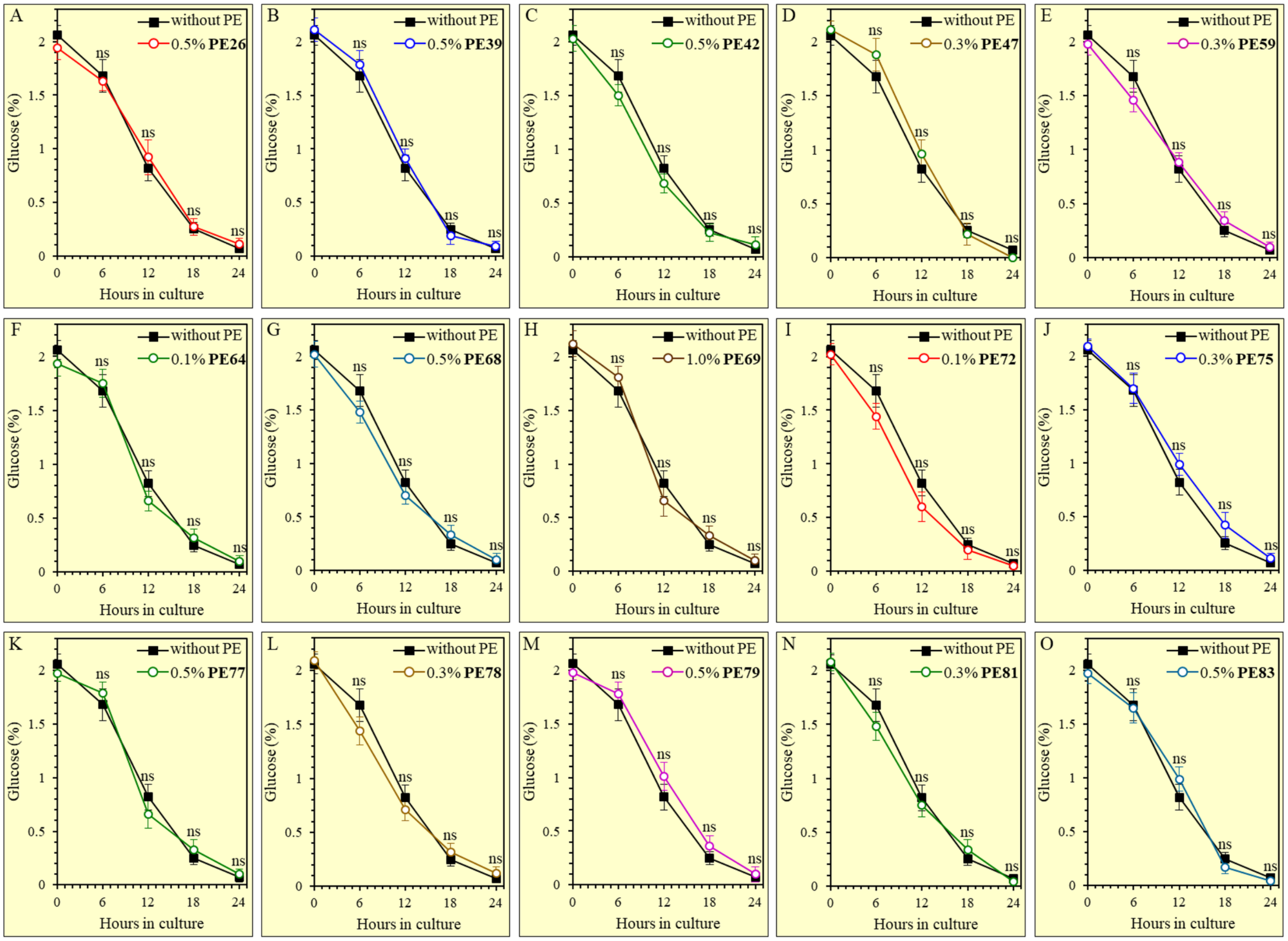

Supplementary Figure 8: None of the fifteen longevity-extending PEs statistically significantly affects glucose consumption by WT yeast cultured under non-CR conditions on 2% (w/v) glucose. WT cells were cultured in the synthetic minimal YNB medium initially containing 2% (w/v) glucose, in the presence of a longevity-extending PE (which was added at an optimal longevity-extending concentration) or its absence. Glucose concentration in the extracellular medium was measured as described in Materials and Methods. Data are presented as means ± SEM (*n* = 3; ns, not significant).

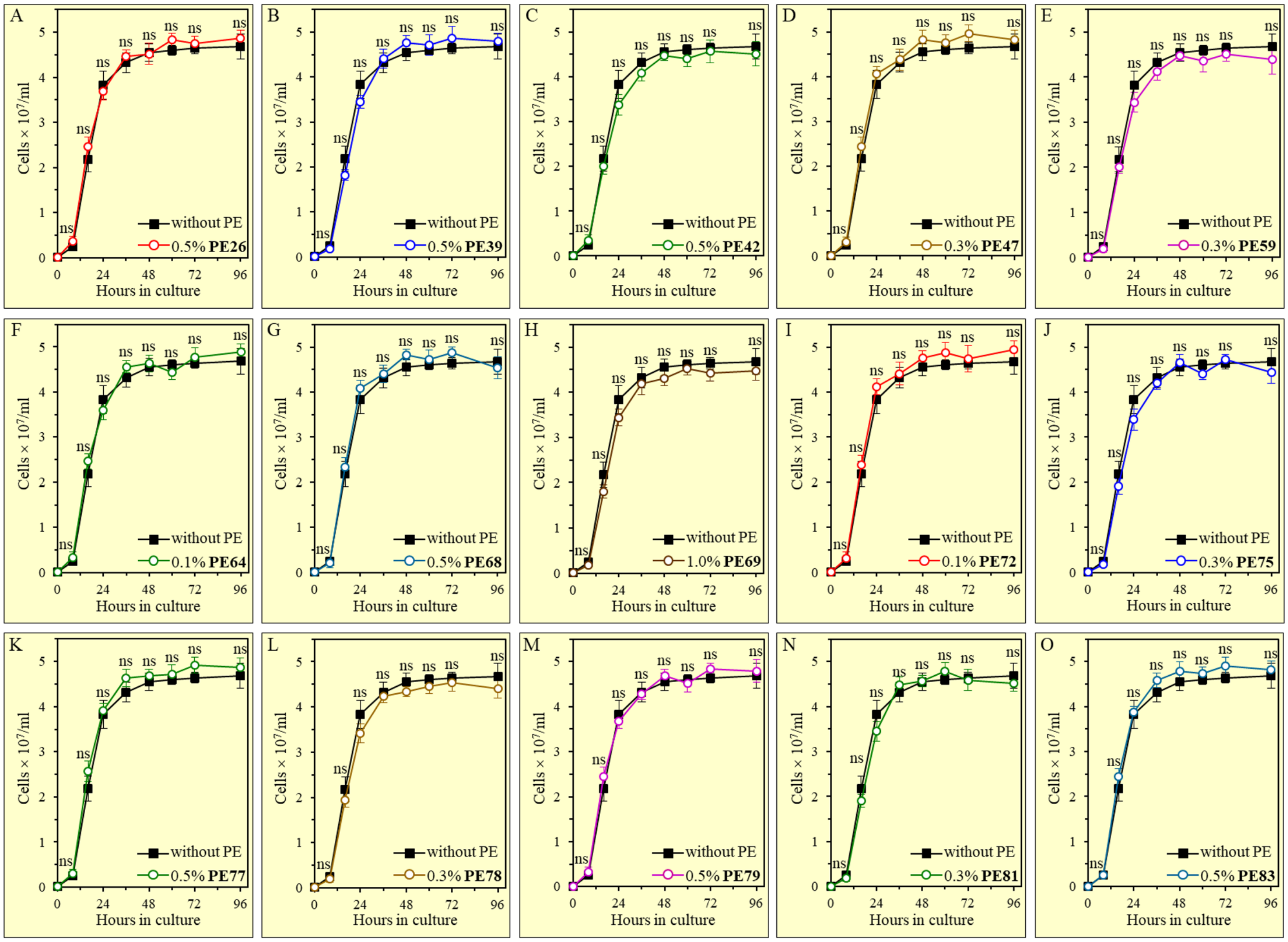

Supplementary Figure 9: None of the fifteen longevity-extending PEs statistically significantly alters the growth rate and maximum cell yield of WT yeast cultures under non-CR conditions on 2% (w/v) glucose. WT cells were cultured in the synthetic minimal YNB medium initially containing 2% (w/v) glucose, in the presence of a longevity-extending PE (which was added at an optimal longevity-extending concentration) or its absence. Cell number was measured as described in Materials and Methods. Data are presented as means ± SEM (n = 3; ns, not significant).

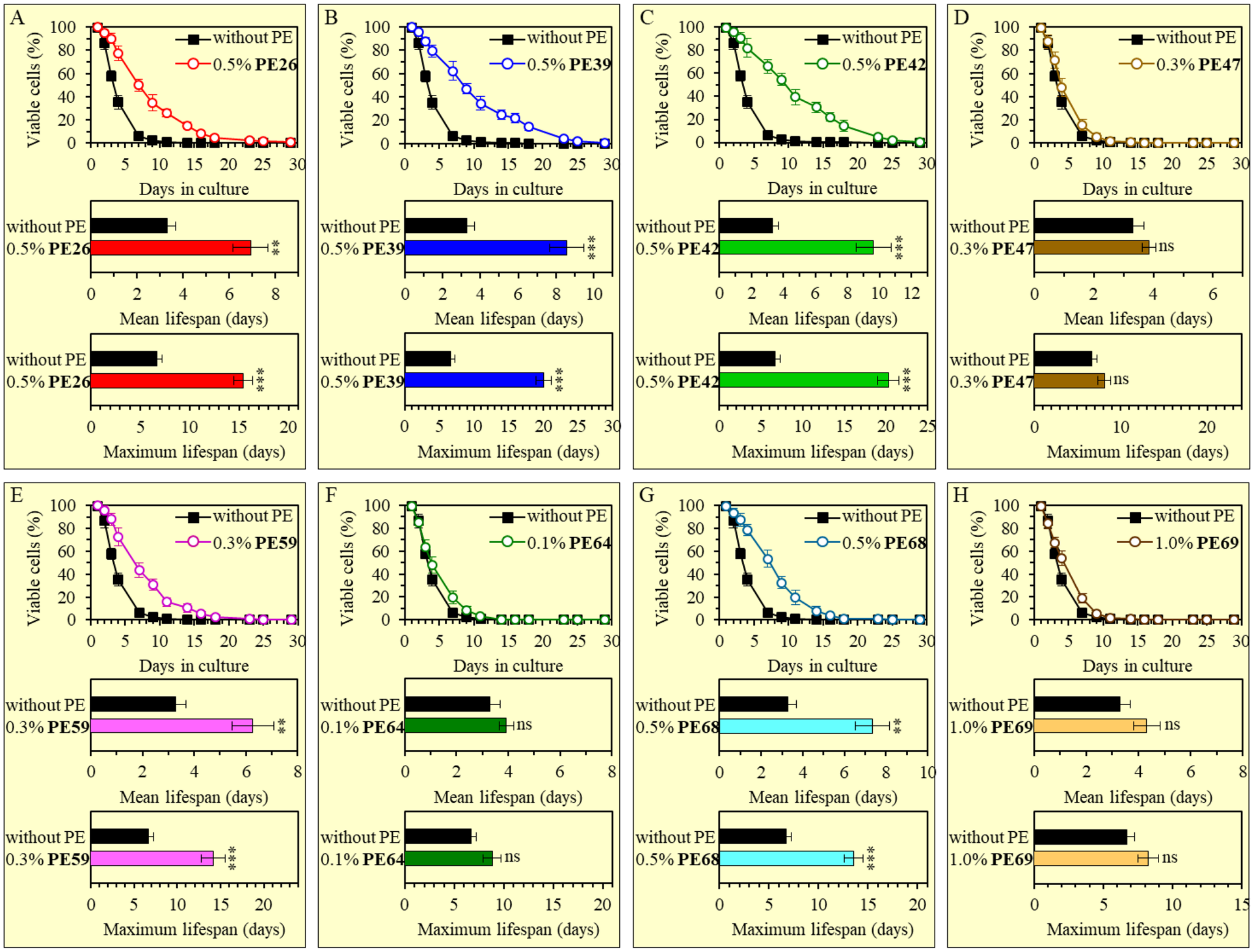

Supplementary Figure 10: 0.5% (w/v) PE26, 0.5% (w/v) PE39, 0.5% (w/v) PE42, 0.3% (w/v) PE59 and 0.5% (w/v) PE68 (but not 0.3% (w/v) PE47, 0.1% (w/v) PE64 or 1.0% (w/v) PE69) extend the CLS of WT yeast cultured under CR conditions on 0.5% (w/v) glucose. WT cells were cultured in the synthetic minimal YNB medium initially containing 0.5% (w/v) glucose, in the presence of a PE or its absence. In the cultures supplemented with a PE, ethanol was used as a vehicle at a final concentration of 2.5% (v/v). In the same experiment, WT cells were also subjected to ethanol-mock treatment by being cultured in the synthetic minimal YNB medium initially containing 0.5% (w/v) glucose and 2.5% (v/v) ethanol. Survival curves (the upper panels in A–H) and the mean and maximum lifespans (the lower two panels in **A–H**) of chronologically aging WT cells cultured without a PE (cells were subjected to ethanol-mock treatment) or with a PE (which was added at the concentration optimal for CLS extension under non-CR conditions) are shown. Data are presented as means ± SEM ($n = 6$). In the upper panels in A–C, E and F, CLS extension was significant for each of the PEs tested ($p < 0.05$; the p values for comparing each pair of survival curves were calculated using the logrank test as described in Materials and Methods). In the lower two panels in A–C, E and F, $^{**}p < 0.01$, $^{***}p < 0.001$; the p values for comparing the means of two in groups were calculated using an unpaired two-tailed t test as described in Materials and Methods). In the upper panels in D, F and H, CLS extension was statistically not significant for each of the PEs tested (the p values for comparing each pair of survival curves were calculated using the logrank test as described in Materials and Methods). In the lower two panels in D, F and H, ns, not significant; the p values for comparing the means of two in groups were calculated using an unpaired two-tailed t test as described in Materials and Methods). Data for mock-treated WT cells are replicated in graphs A–H of this Supplementary Figure and graphs A–G of Supplementary Figure 11.

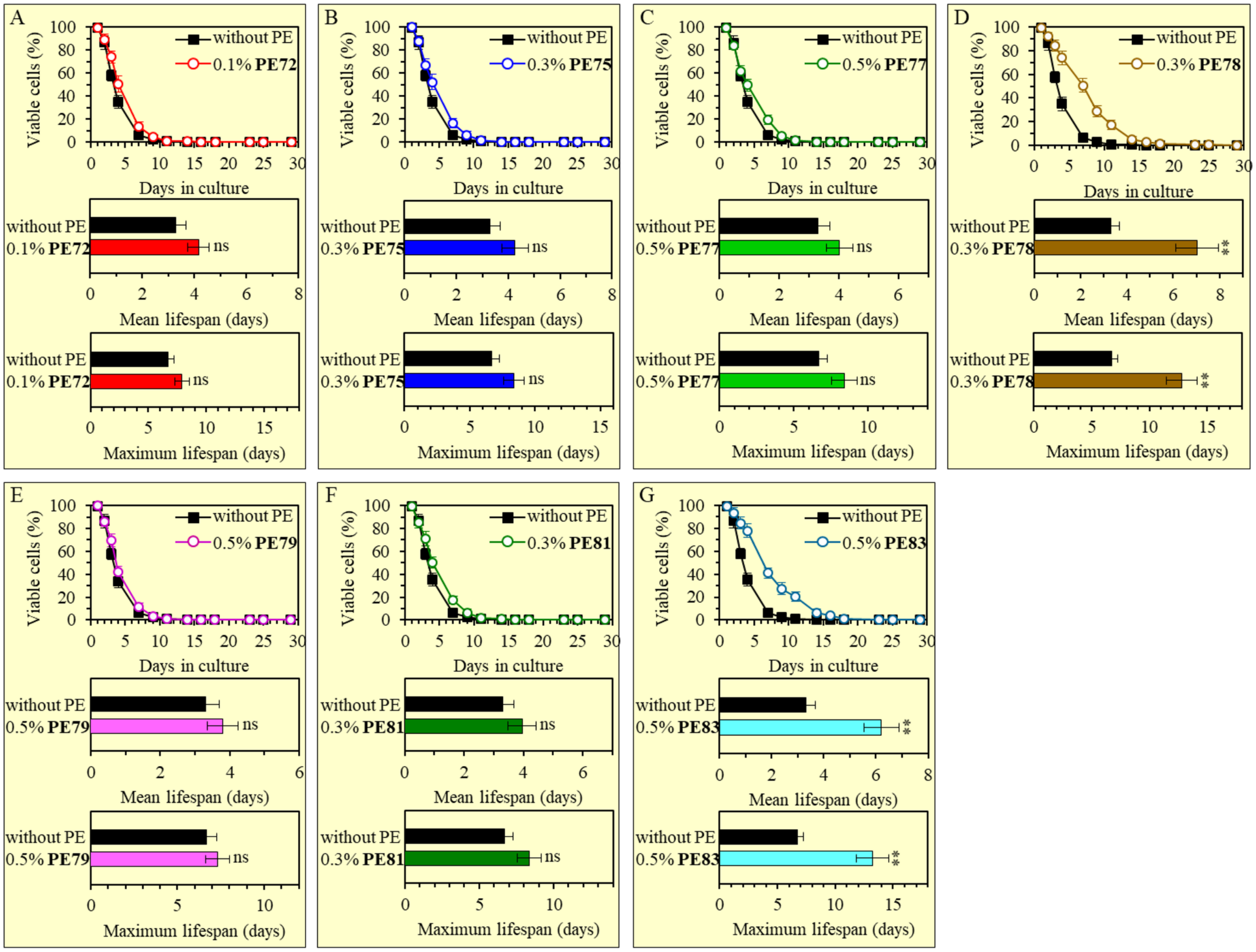

Supplementary Figure 11: 0.3% (w/v) PE78 and 0.5% (w/v) PE83 (but not 0.1% (w/v) PE72, 0.3% (w/v) PE75, 0.5% (w/v) PE77, 0.5% (w/v) PE79 or 0.3% (w/v) PE81) extend the CLS of WT yeast cultured under CR conditions on 0.5% (w/v) glucose. WT cells were cultured in the synthetic minimal YNB medium initially containing 0.5% (w/v) glucose, in the presence of a PE or its absence. In the cultures supplemented with a PE, ethanol was used as a vehicle at a final concentration of 2.5% (v/v). In the same experiment, WT cells were also subjected to ethanol-mock treatment by being cultured in the synthetic minimal YNB medium initially containing 0.5% (w/v) glucose and 2.5% (v/v) ethanol. Survival curves (the upper panels in **A**–**G**) and the mean and maximum lifespans (the lower two panels in A–G) of chronologically aging WT cells cultured without a PE (cells were subjected to ethanol-mock treatment) or with a PE (which was added at the concentration optimal for CLS extension under non-CR conditions) are shown. Data are presented as means ± SEM ($n = 6$). In the upper panels in D and G, CLS extension was significant for each of the PEs tested ($p < 0.05$; the p values for comparing each pair of survival curves were calculated using the logrank test as described in Materials and Methods). In the lower two panels in D and G, $^{**}p < 0.01$; the p values for comparing the means of two in groups were calculated using an unpaired two-tailed t test as described in Materials and Methods). In the upper panels in A–C, E and F, CLS extension was statistically not significant for each of the PEs tested (the p values for comparing each pair of survival curves were calculated using the logrank test as described in Materials and Methods). In the lower two panels in A–C, E and, ns, not significant; the p values for comparing the means of two in groups were calculated using an unpaired two-tailed t test as described in Materials and Methods). Data for mock-treated WT cells are replicated in graphs A–G of this Supplementary Figure and graphs A–H of Supplementary Figure 10.
