# Supplementary material for: Discovery of fifteen new geroprotective plant extracts and identification of cellular processes they affect to prolong the chronological lifespan of budding yeast
Source: Oncotarget. 2020 Jun 9;11(23):2182–203. doi: 10.18632/oncotarget.27615 (PMC7289529; doi:10.18632/oncotarget.27615)
Supplement: Supplementary file 2 [file oncotarget-11-2182-s002.docx]

**Supplementary Table 1: Properties of plant extracts (PEs) used to conduct a new screen for PEs that can prolong the longevity of chronologically aging budding yeast**

| **Abbreviated name of a PE** | **The botanical name of a plant** | **Plant part used to make a PE** | **Properties of a PE** | **A commercial source of a PE** |
| --- | --- | --- | --- | --- |
| PE26 | *Serenoa repens* | Berry | Extraction solvent: carbon dioxide. Extract ratio: 15:1. Composition: natural extract (oil) (45-55%), silica (45-55%). | Idunn Technologies |
| PE38 | *Centella asiatica* | Herb | Extraction solvent: alcohol (50-70%), water (30-50%). Extract ratio: (8-12):1. Composition: 10% asiaticoside, 30% total triterpenes. | Idunn Technologies |
| PE39 | *Hypericum perforatum* | Aerial parts | Extraction solvent: ethanol (60-80%), water (20-40%). Extract ratio: (5-10):1. Composition: 0.3% hypericin. | Idunn Technologies |
| PE40 | *Boswellia serrata* | Resin | Extraction solvent: methanol (80%), water (20%). Extract ratio: 20:1. Composition: 65% boswellic acids. | Idunn Technologies |
| PE41 | *Ruscus aculeatus* | Root | Extraction solvent: ethanol (70-80%), water (20-30%). Extract Ratio: 8:1. Composition: 10% ruscogenins. | Idunn Technologies |
| PE42 | *Ilex paraguariensis* | Leaf | Extraction solvent: water. Extract ratio: (3-10):1. Composition: 2% caffeine. | Idunn Technologies |
| PE43 | *Schisandra chinensis* | Berry | Extraction solvent: ethanol (30%), water (70%). Extract ratio: 4/1, 1% schizandrins. | Idunn Technologies |
| PE44 | *Cynara scolymus L.* | Leaf | Extraction solvent: water. Extract ratio: 4:1. Composition: > 5% cynarin. | Idunn Technologies |
| PE45 | *Allium cepa L.* | Bulb skin | Extraction solvent: ethanol (70%), water (30 %). Extract ratio: (20-25):1. Composition: > 5% quercetin glycoside derivates. | Idunn Technologies |
| PE46 | *Matricaria recutita L.* | Flower | Extraction solvent: ethanol (80%), water (20%). Extract ratio: 5:1. Composition: 3% apigenins. | Idunn Technologies |
| PE47 | *Ocimum tenuiflorum* | Leaf | Extraction solvent: ethanol (90%), water (10%). Extract ratio: 10:1. Composition: > 5% ursolic acid. | Idunn Technologies |
| PE48 | *Rhaphanus sativus L. var. niger* | Root | Extraction solvent alcohol (60-80%), water (40-20%). Extract ratio: 4:1. Composition: unknown. | Idunn Technologies |
| PE49 | *Rosmarinus officinalis L.* | Leaf | Extraction solvent: acetone. Extract ratio: (35-50):1. Composition: > 50% carnosic acid. | Idunn Technologies |
| PE50 | *Angelica archangelica L.* | Root | Extraction solvent: ethanol (50%), water (50%). Extract ratio: 4:1. Composition: > 3% organic acids. | Idunn Technologies |
| PE51 | *Epimedium grandiflorum* | Herb | Extraction solvent: ethanol (60%), water (40%). Extract ratio: 20:1. Composition: 20% icariin. | Idunn Technologies |
| PE52 | *Bacopa monnieri* | Leaf | Extraction solvent: aqueous alcohol. Extract ratio: 10:1. Composition: 20% bacosides. | Idunn Technologies |
| PE53 | *Phaseolus vulgaris* | Bean | Extraction solvent: aqueous alcohol. Extract ratio: 10:1. Composition: unknown. | Idunn Technologies |
| PE54 | *Allium sativum L.* | Bulb | Extraction solvent: water. Extract ratio: 120:1. Composition: 4.5% alliin. | Idunn Technologies |
| PE55 | *Morus alba* | Leaf | Extraction solvent: ethanol (70%), water (30%). Extract ratio: 4:1. Composition: 1% 1-deoxynojirimycin. | Idunn Technologies |
| PE56 | *Saphora Japonica* | Flower | Extraction solvent: ethanol, water. Extract ratio: unknown. Composition: rutin (40%), quercetin (60%). | Idunn Technologies |
| PE57 | *Morus nigra* | Fruit | Extraction solvent: ethanol, water. Extract ratio: 4:1. Composition: unknown. | Idunn Technologies |
| PE58 | *Magnolia officinalis* | Bark | Extraction solvent: unknown. Extract ratio: (35-40):1. Composition: 40% honokiol. | Idunn Technologies |
| PE59 | *Solidago virgaurea* | Herb | Extraction solvent: ethanol (30%), water (70%). Extract ratio: 4:1. Composition: > 2% flavonoid hyperosides. | Idunn Technologies |
| PE60 | *Astragalus membranaceus* | Root | Extraction solvent: ethanol, water. Extract ratio: 8:1. Composition: 16% polysaccharides. | Idunn Technologies |
| PE61 | *Lepidium meyenii* | Root | Extraction solvent: water, then ethanol (96%) and water (4%). Extract ratio: (22-27):1. Composition: 0.6% macamides and macaenes. | Idunn Technologies |
| PE62 | *Taraxacum officinale* | Leaf | Extraction solvent: ethanol (70-80%), water (20-30%). Extract ratio: (4-7):1. Composition: 3% vitexin. | Idunn Technologies |
| PE63 | *Taraxacum officinale* | Root | Extraction solvent: ethanol (60%), water (40%). Extract ratio: 15:1. Composition: 0.3-0.4% phenolic acids (chicoric, chlorogenic and  caftaric acids). | Idunn Technologies |
| PE64 | *Citrus sinensis* | Fruit | Extraction solvent: unknown. Extract ratio: unknown. Composition: ≥ 20% limonene. | Idunn Technologies |
| PE65 | *Piper methysticum* | Root | Extraction solvent: ethanol (65%), water (35%,). Extract ratio: 8:1. Composition: > 30% kavalactones. | Idunn Technologies |
| PE66 | *Handroanthus chrysotrichus* | Bark | Extraction solvent: ethanol (70%), water (30%). Extract ratio: (9-15):1. Composition: unknown. | Idunn Technologies |
| PE67 | *Euterpe oleracea* | Fruit | Extraction solvent: water. Extract ratio: 20:1. Composition: > 10% polyphenols. | Idunn Technologies |
| PE68 | *Humulus lupulus* | Whole plant | Extraction solvent: unknown. Extract ratio: (5.5-6.5):1. Composition: unknown. | Idunn Technologies |
| PE69 | *Vitis vinifera* | Grape skin | Extraction solvent: ethanol (30%), water (70%). Extract ratio: 450:1. Composition: ≥ 5% trans-resveratrol. | Idunn Technologies |
| PE70 | *Vitis vinifera* | Grape | Extraction solvent: water (4%), ethanol (96%). Extract ratio: 200:1. Composition: ≥ 20% oligostilbenes. | Idunn Technologies |
| PE71 | *Malus domestica + Vitis vinifera* | Grape + Fruit | Extraction solvent: water (5%), ethanol (95%). Extract ratio: (500-600):1. Composition: ≥ 95% polyphenols. | Idunn Technologies |
| PE72 | *Andrographis paniculata* | Whole plant | Extraction solvent: unknown. Extract ratio: unknown. Composition: ≥ 20% andrographolides. | Idunn Technologies |
| PE73 | *Oryza sativa* fermented with *Monascus purpureus* yeast | Fermented rice | Extraction solvent: unknown. Extract ratio: unknown. Composition: ≥ 20% monacolin K. | Idunn Technologies |
| PE74 | *Melissa officinalis* | Leaf | Extraction solvent: unknown. Extract ratio: 4:1. Composition: ≥ 1% rosmarinic acid. | Idunn Technologies |
| PE75 | *Hydrastis canadensis* | Root | Extraction solvent: ethanol (75%), water (25%). Extract ratio: (5-7):1. Composition: ≥ 5% berberine and other alkaloids. | Idunn Technologies |
| PE76 | *Polygonum cuspidatum* | Root | Extraction solvent: unknown. Extract ratio: unknown. Composition: ≥ 20% resveratrol. | Idunn Technologies |
| PE77 | *Trigonella foenum-graecum* | Seed | Extraction solvent: ethanol (60%), water (40%). Extract ratio: (5-8):1. Composition: 50% saponins. | Idunn Technologies |
| PE78 | *Berberis vulgaris* | Root bark | Extraction solvent: ethanol (50%), water (50%). Extract ratio: (10-12):1. Composition: 6% berberine. | Idunn Technologies |
| PE79 | *Crataegus monogyna* | Leaf, flower and stem | Extraction solvent: ethanol (80%), water (20%). Extract ratio: (3-6):1. Composition: 1.5% flavonoids. | Idunn Technologies |
| PE80 | *Sophora japonica L.* | Flower bud | Extraction solvent: water. Extract ratio: (16-20):1. Composition: 95% quercetin. | Idunn Technologies |
| PE81 | *Taraxacum erythrospermum* | Leaf | Extraction solvent: ethanol (70-80%), water (20-30%). Extract ratio: (4-7):1. Composition: 3% vitexin. | Idunn Technologies |
| PE82 | *NA* | NA | Na-RALA Powder, Sodium R-lipoate (> 80 % Total R-lipoic Acid) from synthesis. | Idunn Technologies |
| PE83 | *Ilex paraguariensis* | Whole plant | Extraction solvent: unknown. Extract ratio: unknown. Composition: unknown. | Idunn Technologies |
| PE84 | *Vitis vinifera L.* | Seed | Extraction solvent: ethanol, water. Extract ratio: unknown. Composition: 95% polyphenols. | Idunn Technologies |
| PE85 | *Ganoderma lucidum* | Mushroom body | Extraction solvent: unknown. Extract ratio: unknown. Composition: unknown. | Idunn Technologies |
| PE86 | *Panax ginseng* | Root | Extraction solvent: unknown. Extract ratio: unknown. Composition: unknown. | Idunn Technologies |
| PE87 | *Lycium barbarum* | Whole plant | Extraction solvent: unknown. Extract ratio: unknown. Composition: unknown. | Idunn Technologies |
| PE88 | *Hemerocallis fulva* | Flower | Extraction solvent: unknown. Extract ratio: unknown. Composition: unknown. | Idunn Technologies |
| PE89 | *Curcuma L.* | Root | Extraction solvent: unknown. Extract ratio: unknown. Composition: curcumin solid lipid microparticles to improve absorption. | Idunn Technologies |
